# Identification of kynurenine and quinolinic acid as promising serum biomarkers for drug-induced interstitial lung diseases

**DOI:** 10.1186/s12931-023-02653-6

**Published:** 2024-01-14

**Authors:** Yuchen Sun, Kosuke Saito, Atsuhito Ushiki, Mitsuhiro Abe, Yoshinobu Saito, Takeru Kashiwada, Yasushi Horimasu, Akihiko Gemma, Koichiro Tatsumi, Noboru Hattori, Kenji Tsushima, Kazuhisa Takemoto, Rika Ishikawa, Toshiko Momiyama, Shin-ichiro Matsuyama, Noriaki Arakawa, Hirotoshi Akane, Takeshi Toyoda, Kumiko Ogawa, Motonobu Sato, Kazuhiko Takamatsu, Kazuhiko Mori, Takayoshi Nishiya, Takashi Izumi, Yasuo Ohno, Yoshiro Saito, Masayuki Hanaoka

**Affiliations:** 1 Division of Medicinal Safety Science, National Institute of Health Sciences, 3-25-26 Tonomachi, Kawasaki-ku, Kawasaki, Kanagawa 210-9501 Japan; 2 First Department of Internal Medicine, Shinshu University School of Medicine, 3-1-1 Asahi, Matsumoto, Nagano 390-8621 Japan; 3 Department of Respirology (B2), Graduate School of Medicine, Chiba University, 1-8-1 Inohana, Chuo-ku, Chiba-shi, Chiba 260-8677 Japan; 4 Department of Pulmonary Medicine and Oncology, Graduate School of Medicine, Nippon Medical School, 1-1-5, Sendagi, Bunkyo-ku, Tokyo, 113-8602 Japan; 5 Department of Respiratory Medicine, Hiroshima University Hospital, 1-2-3 Kasumi, Minami-ku, Hiroshima, Hiroshima 734-8551 Japan; 6 Division of General Internal Medicine, Department of Internal Medicine, St. Marianna University School of Medicine, 2-16-1 Sugao, Miyamae-ku, Kawasaki, Kanagawa 216-8511 Japan; 7 Division of Pathology, National Institute of Health Sciences, 3-25-26 Tonomachi, Kawasaki-ku, Kawasaki, Kanagawa 210-9501 Japan; 8 Astellas Pharma Inc., 21, Miyukigaoka, Tsukuba, Ibaraki 305-8585 Japan; 9 Daiichi Sankyo RD Novare Co., Ltd., 1-16-13 Kitakasai, Edogawa-ku, Tokyo, 134-8630 Japan; 10 Kihara Memorial Yokohama Foundation, 1-6 Suehiro-cho, Tsurumi-ku, Yokohama, Kanagawa 230-0045 Japan

**Keywords:** Kynurenine, Quinolinic acid, Kynurenine/tryptophan ratio, Drug-induced interstitial lung diseases, Biomarker, Metabolomics

## Abstract

**Background:**

Drug-induced interstitial lung disease (DILD) is a lung injury caused by various types of drugs and is a serious problem in both clinical practice and drug development. Clinical management of the condition would be improved if there were DILD-specific biomarkers available; this study aimed to meet that need.

**Methods:**

Biomarker candidates were identified by non-targeted metabolomics focusing on hydrophilic molecules, and further validated by targeted approaches using the serum of acute DILD patients, DILD recovery patients, DILD-tolerant patients, patients with other related lung diseases, and healthy controls.

**Results:**

Serum levels of kynurenine and quinolinic acid (and kynurenine/tryptophan ratio) were elevated significantly and specifically in acute DILD patients. The diagnostic potentials of these biomarkers were superior to those of conventional lung injury biomarkers, Krebs von den Lungen-6 and surfactant protein-D, in discriminating between acute DILD patients and patients with other lung diseases, including idiopathic interstitial pneumonia and lung diseases associated with connective tissue diseases. In addition to identifying and evaluating the biomarkers, our data showed that kynurenine/tryptophan ratios (an indicator of kynurenine pathway activation) were positively correlated with serum C-reactive protein concentrations in patients with DILD, suggesting the potential association between the generation of these biomarkers and inflammation. Our in vitro experiments demonstrated that macrophage differentiation and inflammatory stimulations typified by interferon gamma could activate the kynurenine pathway, resulting in enhanced kynurenine levels in the extracellular space in macrophage-like cell lines or lung endothelial cells. Extracellular quinolinic acid levels were elevated only in macrophage-like cells but not endothelial cells owing to the lower expression levels of metabolic enzymes converting kynurenine to quinolinic acid. These findings provide clues about the molecular mechanisms behind their specific elevation in the serum of acute DILD patients.

**Conclusions:**

The serum concentrations of kynurenine and quinolinic acid as well as kynurenine/tryptophan ratios are promising and specific biomarkers for detecting and monitoring DILD and its recovery, which could facilitate accurate decisions for appropriate clinical management of patients with DILD.

**Supplementary Information:**

The online version contains supplementary material available at 10.1186/s12931-023-02653-6.

## Background

Drug-induced interstitial lung disease (DILD) is a lung injury that occurs as an adverse effect to various drugs, such as anti-cancer drugs and antirheumatic drugs [1, 2]. The direct cytotoxic effects of these drugs on pulmonary cells and immune cell-mediated pulmonary injuries likely influences incidence of DILD, although pathological mechanisms are unclear [3, 4].

DILD can be classified into subgroups, including diffuse alveolar damage (DAD), organizing pneumonia (OP), nonspecific interstitial pneumonia (NSIP), and hypersensitivity pneumonitis, based on high-resolution computed tomography (HRCT) [1, 2, 4]. DAD confers high mortality, and DILD patients with DAD do not sufficiently respond to steroid treatments [5, 6]. Even if they recover from DILD, they continue to develop lung fibrosis. OP and NSIP have low mortality but should still be identified early to minimize declines in quality of life and delays in treatment [7, 8]. Diagnosis of DILD supports selection of appropriate treatments for DILD and withdrawal of causative drugs.

HRCT chest scans are the gold standard and the most effective and non-invasive method for DILD diagnosis [9]. However, they require respiratory specialists, and to minimize radiation exposure, HRCT can only be performed infrequently in patients with DILD. The available clinical serum markers for DILD are the mucin-like glycoprotein Krebs von den Lungen-6 (KL-6), and the surfactant protein-D (SP-D), which are produced by type II pneumocytes [10–12]. However, these markers do not distinguish DILD from idiopathic interstitial pneumonias (IIPs) and pulmonary involvement associated with connective tissue disease (CTD) [10, 13–15]. Therefore, there is an urgent need for better serum biomarkers that can discriminate DILD from other lung diseases, and monitor exacerbations and recovery.

Recent metabolomic analytical methods enable examination of many metabolites and screening of metabolites specifically responding to disease conditions [16, 17]. The metabolomic profile also provides a comprehensive readout of individual responses to treatment, which may enhance the discovery of biomarkers for the efficacy and adverse effects of drugs [18]. Indeed, several new biomarkers (lysophosphatidylcholines [LPCs]) for DILD were identified in our previous metabolomic study focusing on lipids [19], which showed that LPCs distinguish DILD from IIPs and CTD, but decreased plasma concentrations of LPCs can be observed not only in patients with acute DILD but also those with bacterial pneumonia (BP) [19]. Thus, a novel biomarker specific to DILD needs to be explored.

To this end, we performed metabolomic analysis for hydrophilic molecules using serum samples from patients with DILD to identify new DILD biomarkers. We found and validated that the amount of serum kynurenine (KYN) was elevated in acute DILD patients compared with those in the recovery phase. We also found by quantitative analyses using the combined cohort of DILD patients that serum concentrations of KYN and its downstream metabolite (quinolinic acid [QUNA]), along with KYN/tryptophan (TRP) ratios, can serve as new DILD biomarker candidates. Our data also showed that the KYN/TRP ratios were well correlated with an inflammation biomarker CRP in DILD patients whose blood monocyte levels were significantly increased in acute phase. To explore the biological mechanisms, we investigated the effects of major inflammatory and anti-inflammatory stimuli on the induction of KYN and QUNA in vitro using human monocytic cell lines and their derived macrophage-like cell lines and immortalized lung microvascular endothelial cells (ECs).

## Methods

### Materials

l-KYN and QUNA were purchased from Sigma-Aldrich (St. Louis, MO). l-TRP was purchased from Fujifilm Wako Pure Chemical Corporation (Tokyo, Japan). l-KYN sulfate (ring-D4, 3, 3-D2) (was purchased from Cambridge Isotope Laboratories (Tewksbury, MA). Hippuric acid-d5 were purchased from Toronto Research Chemicals (Toronto, Canada). All other solvents and reagents used were commercially available LC/MS or HPLC grade.

Phorbol 12-myristate 13-acetate (PMA) was obtained from Sigma-Aldrich. The recombinant human cytokines used in this study and their providers were as follows: interferon (IFN)-α 2a and IFNα-2b, PBL Assay Science (Piscataway, NJ); IFNγ and interleukin (IL)-4, Thermo Fisher Scientific (Waltham, MA); IFNβ, IL-1β, and tumor necrosis factor α (TNFα), R&D systems (Minneapolis, MN); IL-6 and IL-10, BioLegend (San Diego, CA). All cytokines were prepared at 10 µg/mL in phosphate-buffered saline (PBS) supplemented with 10% fetal bovine serum (FBS) and stored at − 80 °C.

### Subjects and sample collection

All patient samples were collected from Shinshu University, Nippon Medical School, Chiba University, and Hiroshima University, and healthy controls (HC) were collected from Kitasato University. DILD was diagnosed based on the Japanese diagnostic criteria [2] by respiratory specialists as follows: (1) history of the administration of drugs causing lung injury, (2) appearance of clinical symptoms after drug administration, (3) improvement of the clinical symptoms after discontinuation of the drug, and (4) exclusion when other causes of clinical symptoms were present. Respiratory specialists in each hospital also diagnosed the DILD pattern and recovery [20]. In this study, DILD patients with imaging patterns of DAD or those with other imaging patterns concomitant with the DAD pattern were categorized as DAD/DAD-mixed. The recovery from DILD was checked at least 2 weeks after the onset of DILD, based on the recovery of clinical symptoms and improvement of oxygenation (e.g. SpO_2_). Patients who took DILD-causing drugs without the onset of DILD for at least 12 weeks were recruited as DILD-tolerant patients. Patients with seven other lung diseases, including lung cancer, BP, nontuberculous mycobacteriosis (NTM), IIPs), CTD, chronic obstructive pulmonary disease (COPD), and bronchial asthma (BA), were also recruited as related lung diseases. The basic patient characteristics of the screening cohort, validation cohort, and combined cohort are summarized in Table 1. The biomarker screening cohort was comprised of specimens with enough sample volume for exploratory metabolic study, which were 60 patients with acute DILD and 34 recovered patients collected from February 2016 to July 2018. The independent validation cohort, which comprised 22 patients with acute DILD and 17 recovered patients collected from September 2015 to May 2017, who were not included in the screening cohort, was subjected to quantitative analysis to validate an identified DILD biomarker candidate. Thereafter, the combined cohort was created to evaluate the diagnostic potential of DILD biomarker candidates. The combined cohort was comprised of specimens from the screening and validation cohorts, along with additional samples from one patient with NSIP and two recovered patients with DILD. However, owing to insufficient sample volume, samples from one patient with DAD and one patient with OP in the screening cohort were excluded from the combined cohort. In addition to DILD-related samples, the combined cohort included 20 DILD-tolerant patients, 144 patients with other lung diseases, and 30 healthy donors. Detailed patient backgrounds and measured values of biomarkers for each patient with DILD and the recovered patients are summarized in Additional file 1: Table S1. The median value and range of the number of days between blood sampling in the acute and recovery phases of DILD were 80 days (range 55–98) for the screening cohort and 47 days (range 19–70) for the validation cohort. Detailed information on DILD-tolerant patients, patients with other lung diseases, and healthy donors is summarized in Additional file 1: Table S2.

**Table 1:** Characteristics of all serum samples used in this study

Patient groups	Screening cohort (metabolomic analysis)				Validation cohort (quantitative analysis)				Combined cohort (quantitative analyses)			
	[n]	Ages	BMI	Sex [M/F]	[n]	Ages	BMI	Sex [M/F]	[n]	Ages	BMI	Sex [M/F]
All-DILD	60	70 (66–74)	21.9 (19.5–23.8)	37/23	22	65 (58–71)	22.1 (19.1–23.5)	17/5	81	69 (63–73)	21.9 (19.1–23.7)	53/28
DAD/DAD-mixed	20	68 (64–74)	21.7 (19.9–22.9)	17/3	3	69 (62–70)	22.3 (21.8–22.6)	3/0	22^§^	68 (62–72)	21.7 (20.3–22.6)	19/3
OP	31	69 (66–74)	22.0 (19.0–23.6)	16/15	2	79 (76–82)	21.5 (20.5–22.6)	2/0	32^§^	70 (67–75)	22.0 (19.0–23.6)	18/14
NSIP	9	73 (71–75)	22.5 (20.8–25.3)	4/5	16	63 (59–69)	22.3 (17.9–24.0)	12/4	26^#^	69 (61–73)	22.6 (20.1–24.3)	16/10
Other	–	–	–	–	1	50	20.8	0/1	1	50	20.8	0/1
DILD recovery	34	69 (67–74)	22.7 (20.2–24.8)	22/12	17	62 (55–69)	21.8 (18.2–23.8)	12/5	53^#^	69 (63–72)	22.7 (19.4–24.1)	35/18
DILD-tolerant	–	–	–	–	–	–	–	–	20	70 (67–72)	21.5 (20.2–23.5)	12/8
Lung cancer	–	–	–	–	–	–	–	–	45	72 (62–76)	21.7 (19.8–25.2)	31/14
BP	–	–	–	–	–	–	–	–	14	66 (64–78)	22.8 (19.3–25.0)	11/3
NTM	–	–	–	–	–	–	–	–	15	74 (71–76)	18.4 (17.4–21.7)	5/10
IIPs	–	–	–	–	–	–	–	–	23	73 (62–76)	23.3 (20.4–25.3)	17/6
CTD	–	–	–	–	–	–	–	–	20	68 (65–70)	23.8 (21.7–26.0)	8/12
COPD	–	–	–	–	–	–	–	–	15	70 (65–76)	21.1 (18.8–23.9)	13/2
BA	–	–	–	–	–	–	–	–	12	60 (51–72)	24.5 (20.5–30.2)	3/9
HC	–	–	–	–	–	–	–	–	30	60 (58–62)	20.6 (20.0–22.9)	15/15

The detailed information of each acute DILD patient and the recovered patient can be found in Additional file 1: Table S1. The detailed information of DILD-tolerant patients, patients with other lung diseases, and healthy donors are summarized in Additional file 1: Table S2. The median and range values for age and BMI in each group are shown in the table

^§^One DILD sample from each of DAD/DAD-mixed and OP patient groups was excluded from the combined cohort due to the lack of sample volume available for quantitative analyses

^#^A sample from NSIP patient and two samples from DILD recovery patients were newly collected and included in the combined cohort

M: male; F: female; DAD/DAD-mixed: DILD patients in acute phase with CT pattern of diffuse alveolar damage; OP: DILD patients in acute phase with CT pattern of organizing pneumonia; NISP: DILD patients in acute phase with CT pattern of nonspecific interstitial pneumonia; Other: DILD patients in acute phase with CT pattern other than DAD, OP and NSIP, DILD recovery: patients recovered from DILD; DILD-tolerant: the patient group taking similar medications to DILD group but without DILD onset; BP: bacterial pneumonia, NTM: nontuberculous mycobacteriosis, IIPs: idiopathic interstitial pneumonias, CTD: lung disease associated with connective tissue disease; COPD: chronic obstructive pulmonary disease; BA: bronchial asthma; HC: healthy control

Blood samples were obtained by venipuncture into 10 mL BD Vacutainer® blood collection tubes with a clot activator (BD, Franklin Lakes, NJ). After 60 min incubation at room temperature, the blood samples were centrifuged (1300×*g*, 10 min, room temperature). Serum was then collected and dispensed into screw-capped polypropylene tubes and stored at − 80 °C until metabolite extraction. Serum samples were typically frozen within 2 h (occasionally extended to 4 h at maximum) of blood sampling.

Informed consent was obtained from all the patients in accordance with the Declaration of Helsinki. This study was approved by the Ethics Committees of the National Institute of Health Sciences (NIHS) (Nos. 257 and 259 for NIHS and Nos. 261 and 263 for Kihara Memorial Yokohama Foundation), Shinshu University (Nos. 3318 and 4716), Nippon Medical School (No. 27-11-514), Chiba University (No. 2265), Hiroshima University (No. E-245), Daiichi Sankyo Co., Ltd (No. 15-0504-00), and Astellas Pharma Inc. (No. 000043).

### Metabolomic analysis using serum samples of patients with DILD and in recovery

Hydrophilic metabolites were extracted from serum samples by mixing 50 µL of human serum, 50 µL of water, and 400 µL of acetonitrile (ACN). Subsequently, 350 µL of the mixtures were transferred onto FastRemover for Protein (GL Sciences, Tokyo, Japan) using a Liquid Handler (Microlab NIMBUS with an MPE2 unit, Hamilton, Reno, NV). The flow-through (200 µL) was further mixed with 200 µL of water:ACN (1:4) and then subjected to solid-phase extraction using a FastRemover C18 (GL Sciences) by the Liquid Handler (Microlab NIMBUS). The flow-through (150 µL) was stored at − 20 °C until the following analysis.

The extracted samples were then comprehensively analyzed using hydrophilic interaction liquid chromatography coupled to time-of-flight mass spectrometry (HILIC/TOF–MS) metabolomics platform, in which ACQUITY UPLC (Waters) and SYNAPT G2 (Waters) were used. The analytical method was slightly modified using the method described in a previous report [21]. Briefly, an Aquity amide column 1.7 µm (2.1 mm × 150 mm) (Waters) was used for metabolite separation. Mobile phases A and B were 100% ACN supplemented with 100% distilled water and 0.1% formic acid, respectively. The mobile phase B gradient was as follows: 1%, 0 to 0.1 min; 1% to 70%, 0.1–7 min; 70% to 1%, 7–7.1 min; 1%, 7.1–10 min. The flow rate was 0.4 mL/min. The sample injection volume was 3.5 µL.

After HILIC separation, hydrophilic metabolites were subjected to MS, operating in electrospray ionization positive-ion and negative-ion modes for high sensitivity with a capillary voltage of 1.5 kV, cone voltage of 30 V, and extraction cone voltage of 5 V. The source and desolvation temperatures were set at 120 °C and 500 °C, respectively. The gas flows of the cone and desolvation were 50 L/h and 800 L/h, respectively. Leucine enkephalin (3 ng/mL) was used as the lock mass (556.2771 for positive-ion mode and 554.2615 for negative-ion mode). The scan range was 50–1000 m/z.

We used a previously described method [22] to process the raw data. The detected peaks were annotated using an online database (HMDB, http://www.hmdb.ca/). The processed data obtained in positive-ion mode are summarized in Additional file 1: Table S3. As we could not detect any DILD biomarker candidate in the data obtained in the negative-ion mode, the data were not shown.

Differences in serum metabolite levels between patients with acute and recovery DILD were investigated using volcano plot analysis, effect size (Hedge’s *g*), and receiver operating characteristic curve (ROC) analysis. The criteria for DILD biomarker candidates were as follows: fold change, > 2 or < 0.5; *p*-value, < 5.30 × 10^−5^ = 0.05/943 [two-tailed Welch’s t-test with Bonferroni correction], *g*-value, > 0.8 or < − 0.8; area under the ROC curve (AUROC), > 0.8.

### Quantitative analyses of KYN pathway metabolites in serum samples collected from HC and patients with DILD or other lung diseases

In the quantitative analyses, TRP and KYN levels were measured using reverse-phase liquid chromatography coupled to a triple quadrupole mass spectrometry  (RP-LC/MS) system, whereas QUNA was measured using the Ion chromatography coupled to high-resolution Orbitrap mass spectrometry (IC/MS) system [23] in targeted approaches. The detailed sample extraction and analytical methods are described in the Supplemental Methods (Additional file 2).

Both quantification methods were appropriately validated with reference to the points to consider in the document on analytical assay validation for biomarkers [24] and guidelines/guidance on analytical assay validation for drugs [25–27]. The quantification methods could measure the three metabolites with high reliability because all validated results fulfilled the acceptance criteria of the guidelines/guidance for drugs.

### Measurement of SP-D and KL-6

The serum levels of SP-D and KL-6 were measured using the SP-D kit YAMASA EIA II (Yamasa Co. Ltd., Chiba, Japan) and the E test TOSOH II (Tosoh Co. Ltd., Tokyo, Japan). Both immunoassay kits were used in accordance with the manufacturers’ instructions.

### Enzyme-linked immunosorbent assay (ELISA) assay for human serum IFNγ

Serum IFNγ concentrations in DILD patients, DILD recovery patients, and HC were quantified using a commercially available human IFNγ Quantikine HS ELISA Kit (R&D Systems) according to the manufacturer’s protocol. The serum samples were diluted by four-fold in the diluent buffer in the kit, and 100 µL of the diluted samples was used in the measurements. The optical absorbance at 450 nm and 570 nm (used for wavelength correction) was measured using a microplate reader (TriStar^2^ S LB942, Berthold Technologies, Baden-Württemberg, Germany).

### Cells and cell culture

Human monocytic THP1 (cell number; JCRB0112, lot number; 02052018) and U937 (cell number; JCRB9021, lot number; 11242017) cells were obtained from the Japanese Collection of Research Bioresources Cell Bank (Osaka, Japan) and cultured in Roswell Park Memorial Institute medium 1640 (RPMI-1640; Wako, Osaka, Japan) containing 10% (v/v) heat-inactivated FBS (Sigma-Aldrich) and antibiotics (50 U penicillin and 50 µg streptomycin; Thermo Fisher Scientific).

Immortalized human lung microvascular endothelial HULEC-5a (cell number; CRL-3244™, lot number; 70,031,959) cells were purchased from ATCC (Manassas, VA) and maintained according to the manufacturer’s instructions. Briefly, HULEC-5a cells were cultured in MCDB131 (without l-glutamine; Thermo Fisher Scientific) supplemented with 10 ng/mL recombinant human epidermal growth factor (Fujifilm Wako), 1 μg/mL hydrocortisone (Sigma-Aldrich), and 10 mM glutamine (Fujifilm Wako).

All cells were grown at 37 °C under a 5% CO_2_ atmosphere.

### Cell differentiation and immune stimulations

THP1 and U937 cells (1 × 10^6^ cells) were seeded in 6-well plates and differentiated into macrophage-like cells (dTHP1 and dU937) by treatment with PMA (Sigma-Aldrich) at concentrations of 10 ng/mL (THP1) and 1 ng/mL (U937) for 48 h, respectively. Undifferentiated THP1 and U937 cells treated with 0.1% (v/v) DMSO were used as control cells.

To analyze the effect of various immune stimuli on the levels of gene expression and metabolites in KYN pathways in differentiated macrophage-like cells, the culture medium of dTHP1 and dU937 cells was replaced with fresh RPMI-1640 medium without PMA, and then supplemented with major inflammatory cytokines (IFNα-2a, IFNα-2b, IFNβ, IFNγ, TNFα, IL-1β, and IL-6) or anti-inflammatory cytokines (IL-4 or IL-10) at a concentration of 10 ng/mL. Cells were treated with 10% (v/v) FBS in PBS as a control. The exposure time was 24 h for all conditions.

Similar experiments were performed using HULEC-5a cells. The cells (0.3 × 10^6^ cells) were seeded into 6-well plates. Twenty-four hours after seeding, the cells were exposed to the same immune stimuli described above. The exposure time of all types of immune stimuli for HULEC-5a cells was 48 h.

### RNA extraction and reverse transcription-quantitative real-time PCR

Total RNA was extracted using ISOGEN with Spin Column (Nippon Gene, Tokyo, Japan). The mRNA contained in 1–2 μg total RNA was reverse transcribed using a High-Capacity cDNA Reverse Transcription Kit with RNase Inhibitor (Thermo Fisher Scientific) according to the manufacturer’s protocol.

All reverse transcription-quantitative real-time PCR (RT-qPCR) measurements were performed using a 7500 Fast Real-Time PCR system (Thermo Fisher Scientific). Amplification assays were performed using PowerUp™ SYBR® Master Mix (Thermo Fisher Scientific), and the primer pairs are listed in Additional file 1: Table S4. The specificity of each primer set was confirmed by melting curve analysis. RT-qPCR quantification was independently performed three times. The mRNA expression levels of the target genes were normalized to those of glyceraldehyde-3-phosphate dehydrogenase (GAPDH). The RT-qPCR conditions were: 95 °C for 20 s, followed by 40 cycles of 95 °C for 3 s, and 60 °C for 30 s.

### Western blotting analysis

Western blotting was performed to analyze indoleamine 2,3-dioxygenase 1 (IDO1) protein expression. THP1 and U937 cells (3 × 10^6^ cells) were seeded in T-25 flasks. After cell differentiation and subsequent treatment with IFNγ as described above, the cells were harvested. HULEC-5a cells (1 × 10^6^ cells) were seeded in T-25 flasks and collected two days after incubation with IFNγ. The harvested cells were lysed in radioimmunoprecipitation assay buffer (Fujifilm Wako) supplemented with a protein inhibitor cocktail (Sigma-Aldrich). Protein concentration was measured by the Pierce™ BCA Protein Assay Kit (Thermo Fisher Scientific), and the lysates were stored at − 80 °C until use. Western blotting was performed as previously described [28]. The loading amounts were 15 µg for dTHP1 and dU937 cells and 10 µg for HULEC-5a cells. The proteins transferred onto a polyvinylidene fluoride membrane were blocked with 5% bovine serum albumin (Fujifilm Wako) in Tris-buffered saline with 0.05% Tween 20 (5% (w/v) BSA/TBS-T). The primary antibodies used were mouse anti-IDO1 monoclonal IgG (2000-fold dilution, UMAB251, OriGene Technologies, Rockville, MD) and mouse anti-GAPDH monoclonal IgG (500-fold dilution, MAB374, Merck Millipore, Burlington, MA). Horseradish peroxidase-conjugated proteins binding to mouse IgGκ light chain immunoglobulins (m-IgGκ BP-HRP, 1000-fold dilution, sc-516102, Santa Cruz Biotechnology, Dallas, TX) were used as an alternative to conventional secondary antibodies. All antibodies were diluted with 5% (w/v) BSA/TBS-T. Proteins were detected by chemiluminescence using ECL™ Prime Western Blotting Detection Reagents (Cytiva, Marlborough, MA) and ImageQuant LAS 4000 mini (Cytiva).

### Measurement of KYN pathway metabolites in the cultured cells and their culture medium

The sample extraction method and analytical parameters for KYN pathway metabolites in cell lysates and culture media of IFNγ-stimulated cell lines are described in the Additional file 2: Supplemental Methods. As with human serum, TRP and KYN levels were measured using the RP-LC/MS system, whereas QUNA was measured using the IC/MS system.

### Statistical analysis

Statistical tests for volcano plot analysis were performed using the two-tailed Welch’s t-test with Bonferroni correction. The effect size was calculated using the Hedge’s *g*-value. Nonparametric comparisons of the differences in the median values were statistically tested using the Mann–Whitney U-test. Statistical differences in mean values were tested using Student’s t-test. In the statistical analyses among multiple groups, Bonferroni correction was applied to adjust the* p*-values. Biomarker potentials were assessed by ROC curve analysis. Correlations between two parameters were analyzed using Pearson’s correlation analysis. Statistical analyses were performed using GraphPad Prism 9 (GraphPad Software, San Diego, CA).

## Results

### Metabolomic screening analysis of serum samples obtained from DILD and recovered patients

**Fig. 1:**
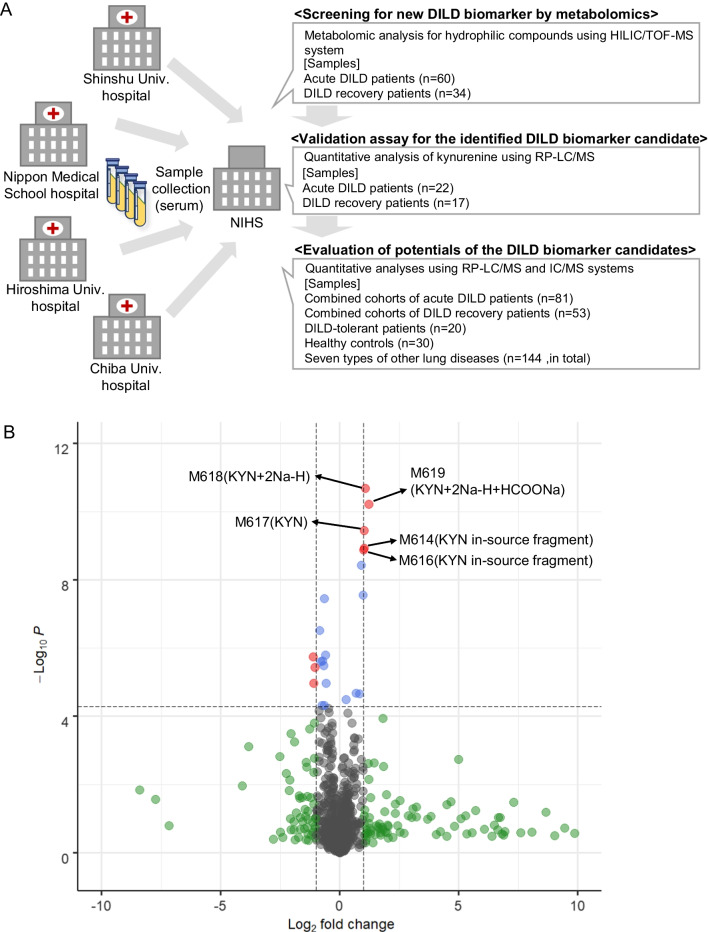
Identification of potential serum DILD biomarkers using metabolomic analyses for hydrophilic molecules. **A** Scheme showing unbiased metabolomic analysis (screening cohort) and quantitative analysis (validation cohort) for hydrophilic molecules in serum obtained from patients with DILD and recovered patients. Subsequently, the potential of the identified DILD biomarker candidates was evaluated using the combined cohort of patients with DILD, along with DILD-tolerant patients, patients with other lung diseases, and HC. Patient characteristics are summarized in Table 1, Additional file 1: Tables S1, and S2. **B** Serum levels of 943 hydrophilic molecules detected in metabolomic analysis were compared between patients with DILD and recovered patients (n = 60 and 34, respectively) in the volcano plot. In the analysis, a very stringent criterion was applied (*p*-value < 5.30 × 10^−5^ = 0.05/943; two-tailed Welch’s t-test with Bonferroni correction). Significantly increased or decreased molecules (fold change > 2 or < 0.5, and *p*-value < 5.30 × 10^−5^) are colored with red. Molecules not-significantly increased or decreased (fold change > 2 or < 0.5, *p*-value > 5.30 × 10^−5^) are colored with green. Only five detected molecules showed significantly increased (*p*-value < 5.30 × 10^−5^, fold change > 2) serum levels with high *g*-value (> 0.8) and high diagnostic potential (area under the ROC curve > 0.8) in acute DILD patients. Those molecules are labeled with tentative metabolite names

To identify new biomarkers for DILD, we performed HILIC/TOF–MS-based non-targeted metabolomic analysis focusing on hydrophilic molecules. Serum samples obtained from patients with DILD in the acute phase (n = 60), including DAD/DAD-mixed (n = 20), OP (n = 31), and NSIP (n = 9), and those from patients with DILD in the recovery phase (n = 34) were extracted and analyzed (Additional file 1: Table S1 and Fig. 1A). Volcano plot analysis revealed that five peaks and three peaks among 943 peaks were significantly increased (*p*-value < 5.30 × 10^−5^ = 0.05/943; two-tailed Welch’s t-test with Bonferroni correction, fold change > 2) and decreased (*p*-value < 5.30 × 10^−5^, fold change < 0.5) in the serum of DILD patients in the acute phase compared to those of recovered patients (Fig. 1B). The DILD biomarker candidates were further narrowed down using the *g*-value (> 0.8, or < -0.8) and area under the ROC curves (> 0.8), yielding five peaks. All were annotated as KYN-derived peaks (Fig. 1B).

### Validation of the elevation of serum KYN concentrations in patients with acute DILD

We collected serum from patients with DILD in the acute phase (n = 22) and recovery phase (n = 17) as a validation set and measured the serum KYN concentration by quantitative analysis. The median KYN concentration in patients with acute DILD was significantly higher than that in recovered patients (3.1 µM vs. 2.3 µM, *p*-value < 0.05, Mann–Whitney U-test, Additional file 3: Fig. S1). This is consistent with the result of the metabolomic study and supports the potential of KYN as a DILD biomarker.

### Evaluation of serum concentrations of KYN pathway metabolites as new DILD biomarkers

**Fig. 2:**
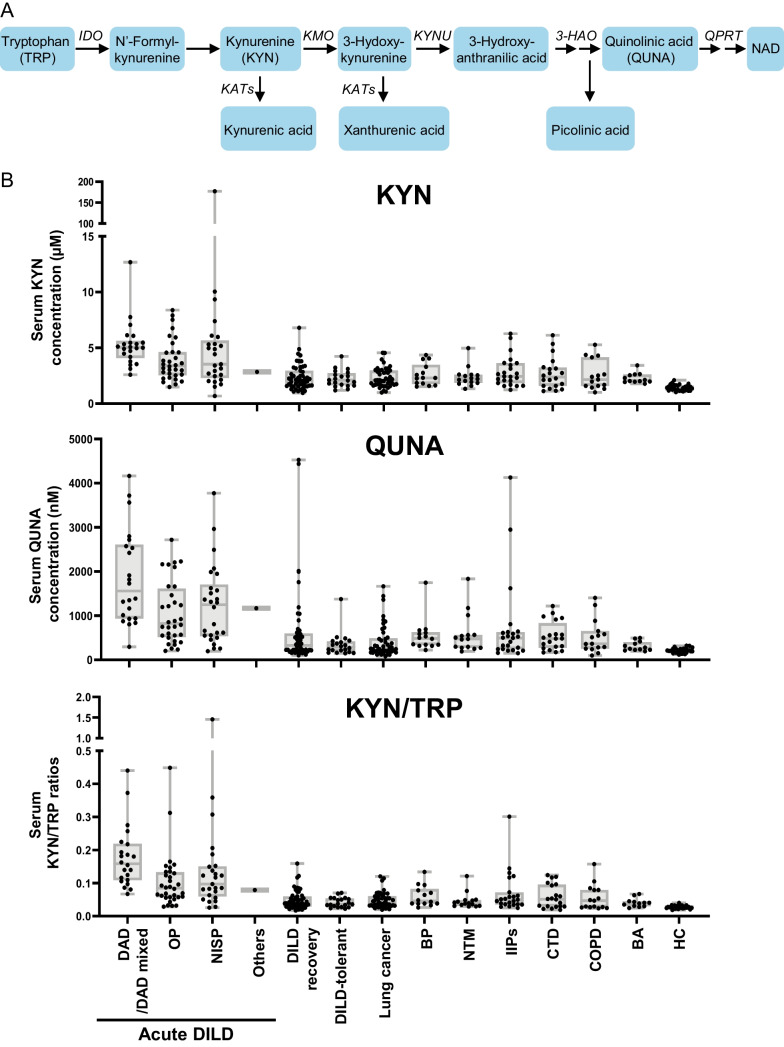
Serum KYN and QUNA concentrations and KYN/TRP ratio in DILD, other lung diseases, and HC. **A** The KYN pathway. Major metabolic enzymes are written in italics. IDO, indoleamine 2,3-dioxygenase; KATs, kynurenine aminotransferases; KMO, kynurenine 3-monooxygenase; KYNU kynureninase, 3-HAO; 3-hydroxyanthranilate oxidase; QPRT, quinolinate phosphoribosyl transferase. **B** Serum concentrations of KYN and QUNA, and KYN/TRP ratio among sample groups are shown as box-and-whisker plots. The middle lines represent the median values and the whiskers represent the highest and lowest values. The results of the statistical comparisons are summarized in Table 2. DAD/DAD-mixed: DILD patients in acute phase with CT pattern of diffuse alveolar damage, OP: DILD patients in acute phase with CT pattern of organizing pneumonia, NISP: DILD patients in acute phase with CT pattern of nonspecific interstitial pneumonia: Other: DILD patients in acute phase with CT pattern other than DAD, OP and NSIP, DILD recovery: patients recovered from DILD, DILD-tolerant: the patient group taking similar medications to DILD group but without DILD onset, BP: bacterial pneumonia, NTM: nontuberculous mycobacteriosis, IIPs: idiopathic interstitial pneumonias, CTD: lung disease associated with connective tissue disease, COPD: chronic obstructive pulmonary disease, BA: bronchial asthma, HC: healthy control

KYN is generated from TRP and metabolized into QUNA (Fig. 2A) via the KYN pathway [29]. According to the previous publication, KYN [1.61 µM], QUNA [0.267 µM], and TRP [64.3 µM] are abundant in the bloodstream of healthy adults, unlike other metabolites of the KYN pathway (0.0258–0.0444 µM, or not detected) [30]. Based on the results of metabolomic screening and quantitative validation tests, we speculated that decreased TRP levels and elevated QUNA levels might also be DILD biomarker candidates. Therefore, in addition to KYN, we also focused on the concentrations of TRP and QUNA in the serum of patients with DILD in a combined cohort, where the combined sample set from screening and validation studies was used (Additional file 1: Table S1). The serum concentrations of KYN and TRP were quantified by RP-LC/MS. The serum concentrations of QUNA were quantified by IC/MS, since QUNA did not give clean peaks in our RP-LC/MS system.

In the combined cohort, KYN, QUNA, and TRP were quantified in serum samples of DILD patients in acute phase (n = 81, including DAD/DAD-mixed [n = 22], OP [n = 32], NSIP [n = 26] and others [n = 1]), DILD patients in recovery phase (n = 53), patients with tolerance (n = 20) and other lung diseases, such as lung cancer (n = 45), BP (n = 14), NTM (n = 15), IIPs (n = 23), CTD (n = 20), COPD (n = 15), BA (n = 12), and healthy controls (HC, n = 30) (Fig. 2B and Additional file 3: Fig. S2). The median values of serum KYN and QUNA levels of all-DILD patients in the acute phase (4.0 µM and 1193.5 nM) were significantly higher than those of the DILD recovery patients (2.1 µM and 325.9 nM, Mann–Whitney U-test with Bonferroni correction) (Fig. 2B and Table 2). Compared with recovered patients, increased KYN and QUNA levels were observed in all types of DILD subgroups (Fig. 2B and Table 2). The median values of serum KYN and QUNA levels tended to be higher in the DAD/DAD-mixed group than other DILD subgroups (KYN, 5.0 µM vs. 3.4–3.5 µM; QUNA, 1557.9 nM vs. 821.0–1246.1 nM), although the only significant differences were observed between the DAD/DAD-mixed and OP groups (Table 2, Mann–Whitney U-test with Bonferroni correction). Statistically significant differences in KYN and QUNA concentrations between acute DILD patients and DILD-tolerant patients (who were administered similar drug(s) but without DILD onset) (KYN, 2.1 µM; QUNA, 286.9 nM) or HC (KYN, 1.4 µM; QUNA, 210.0 nM) were also confirmed (Fig. 2B and Table 2). Serum concentrations of KYN and QUNA in acute DILD patients were higher than those of patients with other lung diseases, including lung cancer (KYN, 2.1 µM; QUNA, 252.3 nM), BP (KYN, 2.3 µM; QUNA, 486.8 nM), NTM (KYN, 2.2 µM; QUNA, 472.2 nM), IIPs (KYN, 2.4 µM; QUNA, 496.4 nM), CTD (KYN, 2.4 µM; QUNA, 480.9 nM), COPD (KYN, 2.2 µM; QUNA, 365.8 nM) and BA (KYN, 2.1 µM; QUNA, 263.5 nM) (Table 2).

**Table 2:** Statistical comparison of serum concentrations of TRP metabolites and TRP/KYN ratio among patient groups

Groups	TRP^#^ (µM)			KYN^#^ (µM)			QUNA (nM)			KYN/TRP^#^(ratio)		
	Median Conc	Adjusted *p*-value		Median Conc	Adjusted *p*-value		Median Conc	Adjusted *p*-value		Median Conc	Adjusted *p*-value	
	(range)	vs DAD/DAD mixed	vs All-DILD	(range)	vs DAD/DAD mixed	vs All-DILD	(range)	vs DAD/DAD mixed	vs All-DILD	(range)	vs DAD/DAD mixed	vs All-DILD
All-DILD	41.3 (30.4–54.0)	–	–	4.0 (2.8–5.4)	–	–	1193.5 (593.6–1910.9)	–	–	0.108 (0.066–0.155)	–	–
DAD/DAD-mixed	32.3 (22.4–40.7)	–	–	5.0 (4.3–5.5)	–	–	1557.9 (955.4–2563.6)	–	–	0.158 (0.111–0.211)	–	–
OP	44.3 (39.2–54.2)	N.S	–	3.4 (2.6–4.6)	**0.03**	–	821.0 (537.3–1512.6)	**0.02**	–	0.085 (0.057–0.131)	**< 0.01**	–
NSIP	41.5 (28.9–59.3)	N.S	–	3.5 (2.5–5.3)	N.S	–	1246.1 (550.2–1609.7)	N.S	–	0.097 (0.063–0.149)	N.S	–
Other	36.1	–	–	2.8	–	–	1165.8	–	–	0.079	–	–
DILD recovery	52.1 (45.7–60.1)	**< 0.01**	**< 0.01**	2.1 (1.6–2.9)	**< 0.01**	**< 0.01**	325.9 (190.0–580.6)	**< 0.01**	**< 0.01**	0.038 (0.029–0.058)	**< 0.01**	**< 0.01**
DILD-tolerant	55.3 (50.3–62.6)	**< 0.01**	**< 0.01**	2.1 (1.7–2.6)	**< 0.01**	**< 0.01**	286.9 (206.7–403.4)	**< 0.01**	**< 0.01**	0.036 (0.030–0.052)	**< 0.01**	**< 0.01**
Lung cancer	50.4 (44.3–57.8)	**< 0.01**	**< 0.01**	2.1 (1.7–3.0)	**< 0.01**	**< 0.01**	252.3 (191.0–474.1)	**< 0.01**	**< 0.01**	0.040 (0.032–0.060)	**< 0.01**	**< 0.01**
BP	45.8 (41.9–52.1)	N.S	N.S	2.3 (1.8–3.2)	**< 0.01**	**< 0.01**	486.8 (341.6–606.1)	**< 0.01**	**< 0.01**	0.046 (0.038–0.076)	**< 0.01**	**< 0.01**
NTM	54.0 (47.1–61.2)	**0.02**	**0.03**	2.2 (1.9–2.6)	**< 0.01**	**< 0.01**	472.2 (283.4–563.9)	**< 0.01**	**< 0.01**	0.039 (0.032–0.048)	**< 0.01**	**< 0.01**
IIPs	47.9 (41.7–60.7)	**0.02**	N.S	2.4 (1.9–3.5)	**< 0.01**	**< 0.01**	496.0 (241.7–620.3)	**< 0.01**	**< 0.01**	0.050 (0.036–0.067)	**< 0.01**	**< 0.01**
CTD	49.3 (41.5–59.3)	**0.01**	N.S	2.4 (1.6–3.2)	**< 0.01**	**0.04**	480.9 (289.2–663.4)	**< 0.01**	**< 0.01**	0.051 (0.030–0.094)	**< 0.01**	**< 0.01**
COPD	51.6 (46.6–57.1)	**0.02**	N.S	2.2 (1.6–3.4)	**< 0.01**	**0.03**	365.8 (266.9–615.8)	**< 0.01**	**< 0.01**	0.047 (0.027–0.079)	**< 0.01**	**< 0.01**
BA	61.0 (53.8–64.5)	** < 0.01**	**< 0.01**	2.1 (2.0–2.6)	**< 0.01**	**< 0.01**	263.5 (226.0–377.4)	**< 0.01**	**< 0.01**	0.039 (0.031–0.044)	**< 0.01**	**< 0.01**
HC	51.4 (47.0–57.4)	** < 0.01**	**< 0.01**	1.4 (1.2–1.6)	**< 0.01**	**< 0.01**	210 (174.8–245.4)	**< 0.01**	**< 0.01**	0.028 (0.025–0.030)	**< 0.01**	**< 0.01**

^#^Samples with missing KYN and TRP concentrations (one in the NSIP and recovery groups, respectively) were excluded from the statistical analyses

Statistical differences between the two groups were examined using the Mann–Whitney U-test with Bonferroni correction. The adjusted *p*-values are listed in table. N.S.: not significant

DAD/DAD-mixed: DILD patients in acute phase with CT pattern of diffuse alveolar damage; OP: DILD patients in acute phase with CT pattern of organizing pneumonia; NISP: DILD patients in acute phase with CT pattern of nonspecific interstitial pneumonia; Other: DILD patients in acute phase with CT pattern other than DAD; OP and NSIP, DILD recovery: patients recovered from DILD; DILD-tolerant: the patient group taking similar medications with DILD group but without DILD onset; BP: bacterial pneumonia; NTM: nontuberculous mycobacteriosis; IIPs: idiopathic interstitial pneumonias; CTD: lung disease associated with connective tissue disease; COPD: chronic obstructive pulmonary disease; BA: bronchial asthma; HC: healthy control

Consistent with the increased serum levels of KYN and QUNA, levels of TRP in all-DILD patients in acute phase (41.3 µM) were significantly lower than those of DILD recovery patients (52.1 µM), DILD-tolerant patients (55.3 µM), patients with lung cancer/NTM/BA (50.4–61.0 µM), and HC (51.4 µM) (Additional file 3: Fig. S2 and Table 2). However, significant differences in TRP levels among all patients with DILD and BP/IIPs/CTD/COPD were not observed (Table 2). The extent of differences in the median values of serum concentrations of TRP between all-DILD patients and other groups were less than those of KYN and QUNA (Table 2), implying that KYN and QUNA might be better DILD biomarkers than TRP.

Additionally, as the increases in KYN and QUNA levels and the reduction of TRP levels were observed synchronously in patients with acute DILD, we compared the KYN/TRP ratio among the groups to investigate the activation of metabolic conversion of TRP via the KYN pathway (Fig. 2B). As with KNY and QUNA levels, the median value of the KYN/TRP ratio in all-DILD patients in the acute phase (0.108) was higher than in those in the DILD recovery group (0.038), tolerant patients (0.036), patients with other lung diseases (0.039–0.051), and HC (0.028) (Fig. 2B, Table 2).

Collectively, our findings demonstrated that serum concentrations of KYN and QUNA were significantly increased in acute DILD patients (especially in acute patients with DAD/DAD-mixed patterns), and that the serum concentration of TRP was decreased in acute DILD patients. Along with the data on raw concentrations of the three metabolites, the elevated KYN/TRP ratio in acute DILD patients suggested that the value of each metabolite and the ratio might be DILD biomarker candidates.

To examine sampling bias, we performed sensitivity analysis for the serum concentrations of KYN pathway metabolites (KYN, QUNA, and TRP) and KYN/TRP ratio between patients with DILD in the acute and recovery phases by dividing the samples into two sub-cohorts by hospital location (Chiba University and Nippon Medical School; Tokyo metropolitan area [sub-cohort A], Shinshu University, and Hiroshima University; the other area [sub-cohort B]). Compared with recovered patients, significant increases in KYN and QUNA levels and the KYN/TRP ratio and a reduction in TRP levels were observed in both subgroups (Additional file 3: Fig. S3, Mann–Whitney U-test). These results indicate that the current findings were not affected by sampling bias.

Next, we asked if the extent of elevated serum KYN and QUNA levels and the KYN/TRP ratio is associated with patient backgrounds, such as specific underlying disease and types of medications in patients with acute DILD. We first tested the effect of the existence of cancers on serum KYN and QUNA levels as well as the KYN/TRP ratio in patients with DILD. The metabolite levels and KYN/TRP ratio between DILD patients with and without cancer were comparable (adjusted *p*-value > 0.05, Mann–Whitney U-test with Bonferroni correction, Additional file 3: Fig. S4A). Next, the association of serum KYN and QUNA levels, as well as the KYN/TRP ratio, with underlying lifestyle-related diseases was investigated. No statistically significant differences among groups were detected between the patients with DILD with or without lifestyle-related diseases (adjusted *p*-value > 0.05, Mann–Whitney U-test with Bonferroni correction, Additional file 3: Fig. S4B). The effect of medication type on the DILD biomarker candidates was also examined. No significant correlation was observed (adjusted *p*-value > 0.05, Mann–Whitney U-test with Bonferroni correction, Additional file 3: Fig. S4C). These results suggest that serum concentrations of KYN, QUNA, and the KYN/TRP ratio are not related to at least the patient characteristics analyzed, indicating that elevated serum KYN and QUNA concentrations as well as the KYN/TRP ratio may be biomarkers for acute DILD patients with a wide range of patient characteristics.

Furthermore, it is intriguing to explore whether the degree of the activation of KYN pathway correlates with the severity or mortality of DILD. Initially, we evaluated the correlation between KYN/TRP ratio and SpO_2_/FiO_2_ ratio, an indicator of the degree of hypoxemia, in DILD in acute phase DILD patients. Our data shown that no statistically significant correlation was observed (r = − 0.1882, *p* = 0.22, Additional file 3: Fig S5A). Additionally, we compared the KYN/TRP ratio between acute DILD patients who survived and those who died due to DILD exacerbation. The result indicated no statistically significant difference in the KYN/TRP ratio between the two groups (Additional file 3: Fig S5B). Collectively, although further investigations are warranted, our findings demonstrate that the activation level of KYN pathway in acute DILD patients may not be associated with its severity and mortality.

### Potential of KYN, QUNA, and KYN/TRP as biomarkers for the diagnosis of the onset of DILD and its recovery

**Fig. 3:**
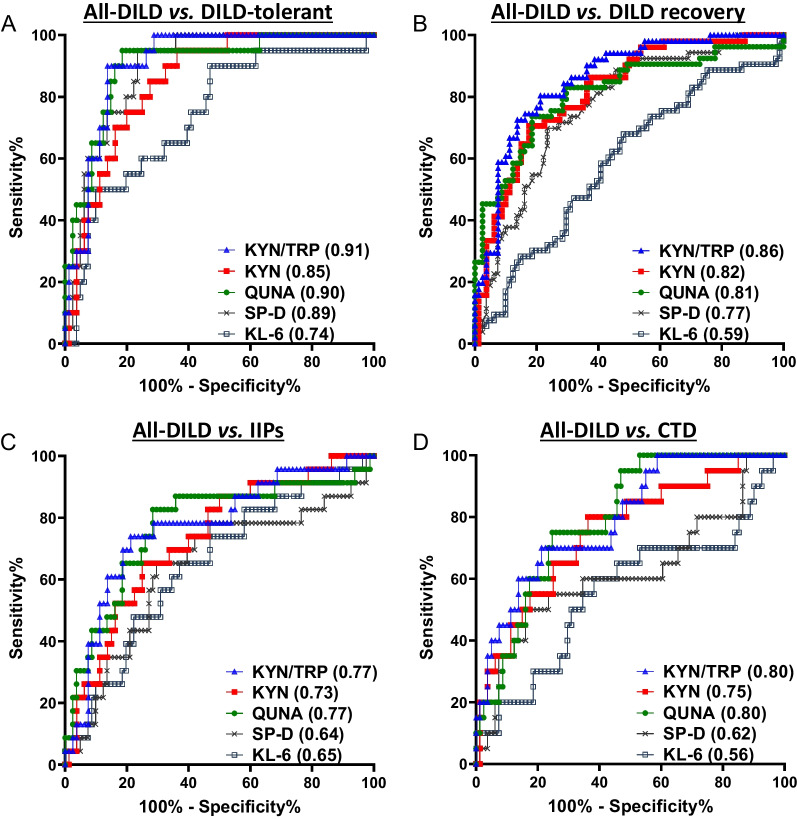
Diagnostic potentials of KYN, QUNA, KYN/TRP ratio, and conventional DILD biomarkers. ROC curve analyses of serum levels of KYN and QUNA, KYN/TRP ratio, and levels of conventional ILD biomarkers (SP-D and KL-6) were performed between the groups using the quantitative data of all samples in the combined cohort. The ROC curves of all-DILD patients compared with DILD-tolerant **A,** DILD recovery **B**, IIPs **C**, or CTD **D** are shown. The values of AUROC are described in the parentheses of the labels for each tested biomarker. The AUROC values for other comparisons are summarized in Table 3

The diagnostic potential of serum KYN, QUNA, and TRP levels and the KYN/TRP ratio for DILD was analyzed using the AUROC values and compared with those of conventional serum markers for interstitial lung diseases (ILD) (KL-6 and SP-D) and inflammation (C-reactive protein [CRP]). The concentration ranges of these markers and results of statistical comparisons are shown in Additional file 3: Fig. S6 and Additional file 1: Table S5, respectively, for all samples. Representative ROC curves for all-DILD patients and DAD/DAD-mixed patients are shown in Fig. 3 and Additional file 3: Fig. S7, respectively. The results of AUROC values are summarized in Table 3.

**Table 3:** Summary of DILD biomarker potentials of TRP metabolites in comparison with conventional serum biomarkers

Comparisons in ROC curve analyses	Diagnostic potentials (AUROC, 95% confidence interval)						
	TRP	KYN	QUNA	KYN/TRP	SP-D	KL-6	CRP
DAD/DAD-mixed vs. DILD recovery	**0.80** (0.68–0.92)	**0.94** (0.89–0.99)	**0.89** (0.81–0.97)	**0.97** (0.93–1.00)	**0.88** (0.79–0.96)	0.66 (0.53–0.78)	**0.88** (0.79–0.96)
DAD/DAD-mixed vs. DILD-tolerant	**0.85** (0.73–0.97)	**0.98** (0.94–1.00)	**0.96** (0.90–1.00)	**1.00** (0.98–1.00)	**0.97** (0.93–1.00)	**0.81** (0.67–0.94)	**0.95** (0.88–1.00)
DAD/DAD-mixed vs. Lung cancer	**0.82** (0.69–0.94)	**0.96** (0.92–1.00)	**0.94** (0.88–1.00)	**0.98** (0.95–1.00)	**0.94** (0.88–0.99)	**0.84** (0.74–0.93)	**0.93** (0.85–1.00)
DAD/DAD-mixed vs. BP	**0.78** (0.62–0.93)	**0.94** (0.87–1.00)	**0.92** (0.82–1.00)	**0.95** (0.88–1.00)	**0.94** (0.86–1.00)	**0.95** (0.87–1.00)	0.63 (0.40–0.85)
DAD/DAD-mixed vs. NTM	**0.85** (0.73–0.98)	**0.96** (0.89–1.00)	**0.90** (0.79–1.00)	**0.98** (0.93–1.00)	**0.84** (0.70–0.98)	**0.88** (0.76–1.00)	**0.88** (0.75–1.00)
DAD/DAD-mixed vs. IIPs	**0.77** (0.63–0.91)	**0.87** (0.77–0.98)	**0.87** (0.76–0.99)	**0.90** (0.80–0.99)	0.59 (0.42–0.76)	0.63 (0.46–0.79)	**0.88** (0.77–0.98)
DAD/DAD-mixed vs. CTD	**0.80** (0.66–0.93)	**0.88** (0.77–0.99)	**0.91** (0.82–1.00)	**0.93** (0.86–1.00)	**0.69** (0.52–0.86)	0.53 (0.35–0.72)	**0.83** (0.69–0.97)
DAD/DAD-mixed vs. COPD	**0.80** (0.66–0.95)	**0.91** (0.81–1.00)	**0.91** (0.82–1.00)	**0.94** (0.87–1.00)	**0.92** (0.84–1.00)	**0.78** (0.61–0.96)	**0.93** (0.86–1.00)
DAD/DAD-mixed vs. BA	**0.89** (0.79–1.00)	**0.99** (0.95–1.00)	**0.98** (0.94–1.00)	**1.00** (0.98–1.00)	**0.99** (0.97–1.00)	**0.96** (0.91–1.00)	**0.98** (0.94–1.00)
DAD/DAD-mixed vs. HC	**0.85** (0.72–0.98)	**1.00** (1.00–1.00)	**1.00** (0.99–1.00)	**1.00** (1.00–1.00)	N.A	N.A	N.A
All-DILD vs. DILD recovery	**0.68** (0.59–0.78)	**0.82** (0.75–0.89)	**0.81** (0.74–0.89)	**0.86** (0.80–0.92)	**0.77** (0.69–0.85)	0.59 (0.49–0.69)	**0.75** (0.65–0.84)
All-DILD vs. DIKLD-tolerant	**0.75** (0.64–0.86)	**0.85** (0.77–0.93)	**0.90** (0.83–0.97)	**0.91** (0.85–0.97)	**0.89** (0.83–0.96)	**0.74** (0.62–0.86)	**0.88** (0.79–0.97)
All-DILD vs. Lung cancer	**0.69** (0.60–0.78)	**0.83** (0.76–0.90)	**0.86** (0.80–0.93)	**0.86** (0.80–0.93)	**0.85** (0.78–0.92)	**0.76** (0.67–0.84)	**0.78** (0.69–0.86)
All-DILD vs. BP	0.59 (0.47–0.72)	**0.79** (0.67–0.90)	**0.80** (0.69–0.91)	**0.79** (0.68–0.90)	**0.86** (0.76–0.95)	**0.88** (0.79–0.96)	0.58 (0.40–0.76)
All-DILD vs. NTM	**0.74** (0.63–0.85)	**0.83** (0.73–0.93)	**0.79** (0.67–0.90)	**0.88** (0.80–0.96)	**0.73** (0.60–0.87)	**0.80** (0.68–0.91)	**0.76** (0.61–0.91)
All-DILD vs. IIPs	**0.65** (0.53–0.77)	**0.73** (0.62–0.84)	**0.77** (0.65–0.89)	**0.77** (0.66–0.88)	**0.64** (0.50–0.78)	**0.65** (0.52–0.77)	**0.68** (0.56–0.80)
All-DILD vs. CTD	**0.66** (0.54–0.78)	**0.75** (0.63–0.87)	**0.80** (0.70–0.89)	**0.80** (0.69–0.90)	0.62 (0.47–0.78)	0.56 (0.41–0.71)	**0.73** (0.58–0.89)
All-DILD vs. COPD	**0.68** (0.55–0.81)	**0.79** (0.66–0.91)	**0.80** (0.69–0.91)	**0.82** (0.70–0.94)	**0.81** (0.70–0.92)	**0.72** (0.56–0.88)	**0.76** (0.64–0.88)
All-DILD vs. BA	**0.82** (0.71–0.93)	**0.85** (0.77–0.93)	**0.93** (0.87–0.98)	**0.91** (0.85–0.97)	**0.94** (0.89–0.99)	**0.89** (0.81–0.96)	**0.92** (0.86–0.98)
All-DILD vs. HC	**0.73** (0.63–0.82)	**0.97** (0.94–1.00)	**0.98** (0.95–1.00)	**0.98** (0.95–1.00)	N.A	N.A	N.A

AUROC values were calculated from ROC curves, and those with statistical significance (*p-*value < 0.05) are indicated in bold letters

DILD recovery: patients recovered from DILD; DILD-tolerant: the patient group taking similar medications to the DILD group but without DILD onset; BP: bacterial pneumonia; NTM: nontuberculous mycobacteriosis; IIPs: idiopathic interstitial pneumonias; CTD: lung disease associated with connective tissue disease; COPD: chronic obstructive pulmonary disease; BA: bronchial asthma; HC: healthy control; N.A.: not applicable

The potential for diagnosing the onset of DILD was evaluated for the identified biomarker candidates via ROC curves analysis, comparing acute DILD to the DILD-tolerant control patients or HC (Fig. 3A and Table 3). The diagnostic potentials (AUROC values) of serum KYN (0.85) and QUNA (0.90) levels and KYN/TRP ratio (0.91) were comparable or superior to those of KL-6 (0.74), SP-D (0.89), and CRP (0.88). Moreover, the AUROC value of TRP between the all-DILD and tolerant groups was 0.75, which was lower than that of KYN, QUNA, and the KYN/TRP ratio (Table 3), indicating that the diagnostic potential of TRP as a DILD biomarker is insufficient. KYN, QUNA, and KYN/TRP also showed high AUROC values (≥ 0.97, Table 3) compared with HC. These data demonstrated the usefulness of the KYN, QUNA, and KYN/TRP in the diagnosis of DILD onset.

As a next step, we assessed their biomarker potentials for diagnosing DILD recovery. ROC analyses showed that the AUROC values between the all-DILD and DILD recovery groups of KYN (0.82) and QUNA (0.81) were higher than those of conventional serum biomarkers (KL-6 [0.59], SP-D [0.77], and CRP [0.75], Fig. 3B and Table 3). In addition, compared with that of KYN, the KYN/TRP ratio showed a slightly improved diagnostic potential (0.86) between the all-DILD and DILD recovery groups (Fig. 3B). Collectively, our results indicate that serum KYN and QUNA levels and the KYN/TRP ratio are feasible biomarkers for monitoring recovery from DILD.

The positivity rate of the biomarker candidates and conventional ILD biomarkers was analyzed (Additional file 1: Table S6). Using Youden’s index of the ROC curve built between patients with acute DILD and those with DILD recovery, the optimal cutoff values of the identified biomarker candidates were determined. The positivity rate of biomarker candidates in patients with acute DILD (78.8–82.5%) was comparable to that of conventional biomarkers (67.9–81.5%). Notably, KYN, QUNA, and KYN/TRP (new biomarkers) exhibited lower positivity rates than conventional biomarkers, not only in DILD-tolerant patients (5.0–30.0% [new biomarkers] vs. 25.0–35.0% [conventional biomarkers] but also in recovered patients (19.6–29.4% [new biomarkers] vs. 45.3%–52.8% [conventional biomarkers]), demonstrating the higher specificity of the new biomarkers.

In addition to the evaluation of biomarker potential in all-DILD patients, our focus extended to DAD/DAD-mixed patients who suffered from more severe DILD than patients with other imaging patterns. In the ROC analyses between the DAD/DAD-mixed and tolerant groups, a strikingly high diagnostic potential of KYN, QUNA, and KYN/TRP was observed (AUROC ≥ 0.96, Additional file 3: Fig. S7A and Table 3). Furthermore, the AUROC values of these biomarker candidates for diagnosing DILD recovery ranged from 0.89 to 0.97 (Additional file 3: Fig. S7B and Table 3). These data suggest that the performance of the novel DILD biomarker candidates tends to be higher in patients with DAD/DAD-mixed DILD than in those with all-DILD.

Meanwhile, the diagnostic potential of the KYN, QUNA, and KYN/TRP for detecting of the onset of DILD and its recovery was also assessed in OP and NSIP patients (Additional file 1: Table S7). While the AUROC values were lower than those observed in DAD/DAD-mixed patients, the AUROC values still exhibited significant diagnostic capability in OP and NSIP patients. These findings suggest the usefulness of these biomarker candidates in OP and NSIP patients.

### Superiority of KYN, QUNA, and KYN/TRP over conventional ILD biomarkers in the differential diagnosis between DILD and other lung diseases, including IIPs and CTD

Discrimination of DILD from IIPs and CTD is important because administration of causative drugs should be stopped in patients with DILD but not in those with IIPs and CTD. The drawbacks of the clinically available ILD biomarkers (KL-6 and SP-D) include their inability to distinguish DILD from IIPs and CTD [10, 13–15]. To overcome these drawbacks, new DILD biomarkers must exhibit higher specificity for DILD, enabling them to effectively differentiate DILD from IIPs and CTD. To determine whether the identified biomarker candidates are DILD-specific biomarkers, we calculated AUROC values in combinations of all-DILD and patients with IIPs or CTD (Fig. 3C and D, Table 3). Higher diagnostic potentials between IIPs patients and all-DILD patients were observed for KYN (0.73), QUNA (0.77), and KYN/TRP ratio (0.77) than for KL-6 (0.65) and SP-D (0.64) (Fig. 3C). As for the diagnostic power to differentiate between DILD and CTD, the new biomarkers had much higher AUROC values (0.75–0.80) than KL-6 (0.56) and SP-D (0.62) (Fig. 3D). Among the new biomarkers, KYN/TRP ratio showed the lowest the positivity rates in patients with IIPs (26.1%) and CTD (35.0%); the rates were much lower than those of KL-6 (70.0–82.6%) and SP-D (50.0–82.6%) (Additional file 1: Table S6). Additionally, a trend toward higher diagnostic performance to distinguish DILD from IIPs and CTD (0.87–0.93 [new biomarkers] vs*.* 0.59–0.69 [conventional biomarkers]) was observed in DAD/DAD-mixed patients (Additional file 3: Fig. S7C and D and Table 3). Similar tendency was also observed in OP and NSIP patients (Additional file 1: Table S7). These data indicate that the new biomarkers have the potential to overcome the weakness of the conventional markers in terms of discriminating DILD from IIPs and CTD.

In the current study, we also compared the diagnostic potential of the new biomarkers for discriminating DILD from other lung diseases, including lung cancer, BP, NTM, COPD, and BA. The AUROC values for the new biomarkers were comparable to those of the ILD biomarkers in the analyses comparing all-DILD with each related-lung disease (0.79–0.93 vs. 0.72–0.94, Table 3). Similar results were observed for DAD/DAD-mixed, OP, and NSIP patients (Additional file 1: Table S7 and Table 3). Taken together, our findings indicate that serum KYN and QUNA levels, as well as the KYN/TRP ratio, are useful biomarkers to support the specific diagnosis of DILD in patients with heterogeneous clinical backgrounds.

### IDO1 induces KYN pathway activation upon macrophage differentiation and IFNγ stimulation in monocytic cell lines

**Fig. 4:**
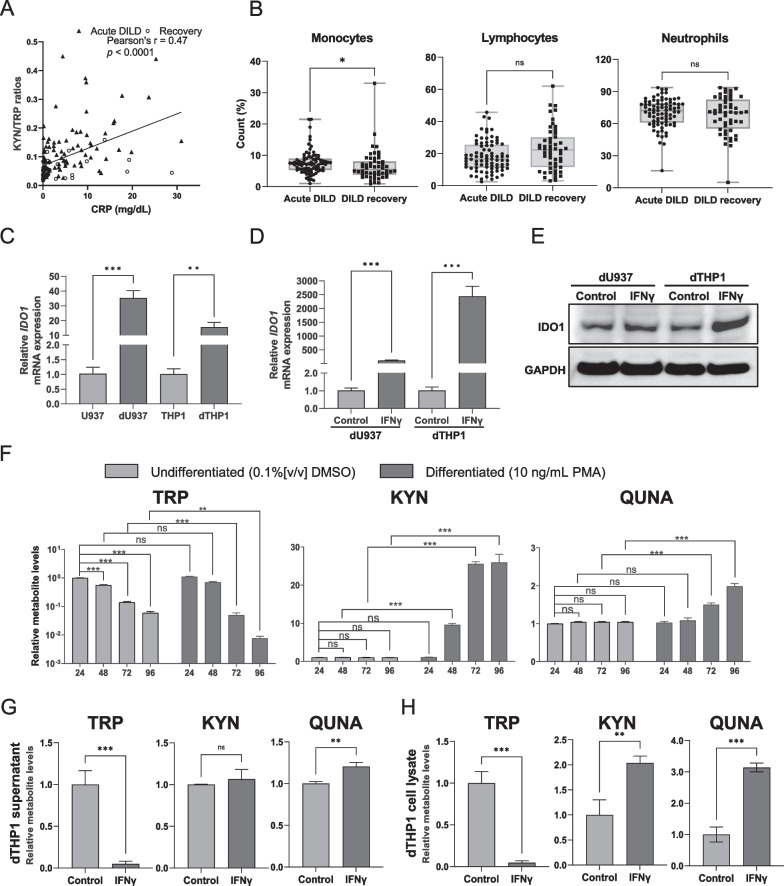
Activation of KYN pathway in monocytic cell lines upon macrophage differentiation and IFNγ stimulation. **A** Correlation analysis of KYN/TRP ratio with serum CRP concentrations. **B** Comparison of median values of the percentage of major white blood cell types in patients with DILD and recovered patients. **C** Expression levels of *IDO1* mRNA were examined using RT-qPCR in undifferentiated (U937 and THP1) cells and differentiated (dU937 and dTHP1) macrophage-like cell lines. **D** Fold changes of *IDO1* mRNA expression levels upon vehicle (10% FBS-PBS) or IFNγ (10 ng/mL) treatment were examined in differentiated macrophage cell lines (dTHP1 and dU937). **E** Western blotting analysis of IDO1 expression in dU937 and dTHP1 cells upon treatment with vehicle (10% FBS-PBS) or IFNγ (10 ng/mL) for 24 h. GAPDH expression was analyzed as a loading control. Uncropped images are shown in Additional file 4. **F** Levels of TRP, KYN, and QUNA were measured in undifferentiated or differentiated THP1 cell supernatant treated with 0.1% (v/v) DMSO or PMA at 10 ng/mL for 24, 48, 72, and 96 h. The metabolite level in undifferentiated THP1 cells after 24 h DMSO exposure was set as 1. The time-dependent changes of each metabolite level in undifferentiated cells and levels between undifferentiated and differentiated THP1 cells were statistically compared. **G** and **H** Relative levels of TRP, KYN, and QUNA in supernatant and whole cell lysate of dTHP1 cells treated with 10% FBS-PBS (control) or IFNγ (10 ng/mL) for 24 h are shown. Each bar represents the mean ± standard deviation of three independent experiments. Statistical significance of mean or median values was tested using Student’s t-test or Mann–Whitney U test (ns, not significant; *p-value < 0.05, **p-value < 0.01; ***p-value < 0.001), respectively. Bonferroni correction was conducted for multiple comparisons

Patients with DILD usually have severe lung inflammation and may show immunological activation. A significant correlation between CRP levels and KYN/TRP ratios was observed in patients with DILD (r = 0.47, *p* < 0.0001, Fig. 4A), suggesting the potential association of inflammation with metabolic activation of the KYN pathway. Next, we compared the percentage of major white blood cell types (monocytes, lymphocytes, and neutrophils) between patients with acute DILD and recovered patients to show the cell types responsible for KYN production. The percentage of monocytes was significantly higher in patients with acute DILD than in recovered patients (7.30% vs. 5.45%, *p* < 0.05, Mann–Whitney U-test, Fig. 4B). Meanwhile, the percentage of lymphocytes and neutrophils between acute and recovery phases was comparable (Fig. 4B). These data suggest that monocytes or cells differentiated from monocytes are involved in KYN pathway activation.

To clarify detailed molecular mechanisms underlying activation of the KYN pathway in patients with DILD, we explored immune-related biological molecules that induce the expression of the rate-limiting enzyme, IDO1, which mediates the conversion from TRP to KYN in the KYN pathway (Fig. 2A) [29, 31]. To date, IDO1 expression and activity in alveolar macrophages have been reported in a mouse model of pneumonia caused by allogeneic hematopoietic stem cell transplantation or viral infection [32, 33]. As monocytes can differentiate into macrophages under inflammatory conditions, we performed in vitro analyses to investigate the effects of macrophage differentiation and immune-stimulations on the induction of IDO1 expression and metabolic changes of KYN, QUNA, and TRP.

First, the effect of monocytes on macrophage differentiation was studied using THP1 and U937 monocytic leukemia cell lines as models. These cell lines differentiate into macrophage-like cells upon stimulation with PMA. To examine IDO1 expression in differentiated macrophages, we measured *IDO1* mRNA expression in THP1 and U937 cell lines with or without PMA treatment (Fig. 4C). The results of RT-qPCR showed more than tenfold induction of *IDO1* mRNA expression can be observed in differentiated THP1 and U937 cells (dTHP1 and dU937) compared with undifferentiated cells, implying that differentiation of monocytes into macrophages contributes to IDO1 induction.

Given that the expression of IDO1 is affected by immunological stimulation [34–36], we examined the effect of various inflammatory stimuli (IFNα-2a, IFNα-2b, IFNγ, IFNβ, TNFα, IL-1β, IL-6) and anti-inflammatory stimuli (IL-4 and IL-10) on *IDO1* mRNA expression in dTHP1 and dU937 cells (Additional file 3: Fig. S8A and B). In both dTHP1 and dU937 cells, levels of *IDO1* mRNA were significantly increased by treatment with IFNα-2a, IFNα-2b, IFNβ, IFNγ, and TNFα (adjusted *p*-value < 0.01, Student’s t-test with Bonferroni correction), whereas its levels were decreased by treatment with anti-inflammatory IL-4 (adjusted *p*-value < 0.01). IL-1β and IL-10 treatment did not change *IDO1* expression in dTHP1 cells, but significantly induced or reduced *IDO1* expression in dU937 cells (adjusted *p*-value < 0.01). IL-6 treatment moderately decreased *IDO1* expression in dTHP1 cells but not in dU937 cells. As IFNγ treatment produced the highest induction of *IDO1* mRNA in differentiated macrophages, we used Western blotting to examine the protein levels in cells with and without IFNγ treatment. The expression of IDO1 was higher in dTHP1 cells treated with IFNγ than in untreated cells (Fig. 4E). The protein expression of IDO1 in dU937 cells was mildly induced by IFNγ treatment (Fig. 4E). Collectively, these results demonstrate that differentiated macrophages can respond to stimulation by various cytokines to produce IDO1 in vitro.

To examine the effects of IDO1 induction on the extracellular secretion of KYN and QUNA upon the differentiation of THP1 cells, we measured TRP, KYN, and QUNA levels in the supernatant of THP1 cells over the time course of PMA treatment (Fig. 4F). TRP levels in the THP1 cell supernatant decreased in a time-dependent manner from 24 to 96 h, even without PMA treatment (adjusted *p*-value < 0.01, Student’s t-test with Bonferroni correction), indicating basal consumption of TRP for cell proliferation. TRP levels in the supernatant of dTHP1 cells were significantly lower than those in undifferentiated THP1 cells after 72 h of incubation (adjusted *p*-value < 0.01, Fig. 4F), implying that the differentiation of THP1 cells enhanced TRP consumption. KYN levels in dTHP1 cells increased in the supernatant of dTHP1 cells as time progressed from 48 to 96 h (9.9- to 25.9-fold, adjusted *p*-value < 0.001 compared with undifferentiated cells, Fig. 4F), whereas KYN levels did not change in undifferentiated THP1 cells. Following the increase in KYN level, QUNA levels in the dTHP1 cell supernatant were significantly increased from 72 h after PMA treatment (adjusted *p*-value < 0.001 compared with undifferentiated cells, Fig. 4F). These results suggest that the differentiation of monocytes to macrophages enhances TRP metabolism via the KYN pathway and leads to the secretion of its metabolites, such as KYN and QUNA, into the extracellular space.

As IFNγ induced IDO1 expression at the highest level in dTHP1 cells (Additional file 3: Fig. S8B), we analyzed the effects of treatment with IFNγ for 24 h on the levels of TRP, KYN, and QUNA in the supernatant of dTHP1 cells. TRP levels in the supernatant of dTHP1 cells were dramatically reduced by IFNγ treatment (*p*-value < 0.001, Fig. 4G). In contrast, KYN levels in the supernatant of dTHP1 cells were not changed by IFNγ treatment (Fig. 4G). Nevertheless, extracellular QUNA levels were increased by IFNγ treatment (*p* < 0.01, Fig. 4G). As clear increases in KYN level were not observed in the supernatants, we also analyzed the levels of TRP metabolites in the whole cell lysates of dTHP1 cells treated with IFNγ (Fig. 4G). Consistent with the findings using supernatants, TRP levels were significantly decreased by IFNγ treatment (*p*-value < 0.001, Fig. 4H). Moreover, a significant elevation in KYN (*p*-value < 0.01) and QUNA (*p*-value < 0.001) levels occurred after IFNγ treatment (Fig. 4H). Similar experiments were performed using dU937 cells (Additional file 3: Fig. S9). IFNγ-based activation of the KYN pathway was also observed in dU937 cells, although the extent of activation differed from that in dTHP1 cells (Additional file 3: Fig. S9).

Taken together, our findings imply that inflammation in the lungs of patients with acute DILD stimulates IFNγ signaling, leading to IDO1 production in macrophages differentiated from monocytes, thereby inducing the metabolism of TRP to KYN and QUNA and contributing to the increase in KYN and QUNA levels in the systemic circulation.

### IFNγ stimulation induces the production of KYN but not QUNA in human lung microvascular ECs

**Fig. 5:**
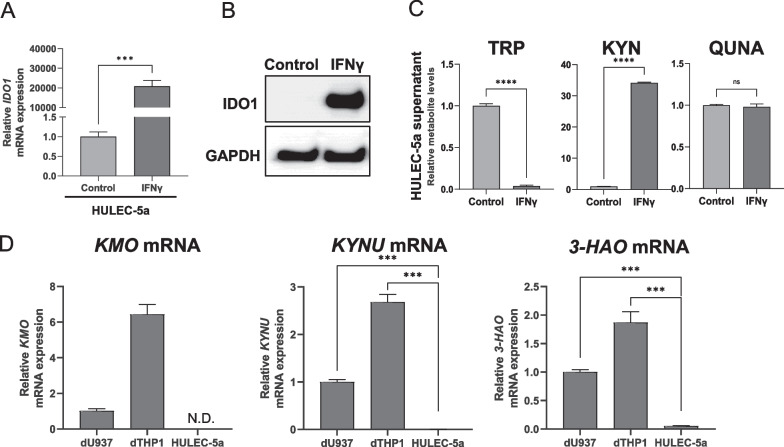
Activation of KYN pathway in human lung ECs upon IFNγ stimulation. **A** Fold changes of *IDO1* mRNA expression levels upon vehicle (10% FBS-PBS) or IFNγ (10 ng/mL) treatment were examined using HULEC-5a cells. **B** Western blotting analysis of IDO1 expression in HULEC-5a cells upon treatment with vehicle or IFNγ (10 ng/mL) for 48 h. GAPDH expression was analyzed as a loading control. Uncropped images are shown in Additional file 5. **C** Relative levels of TRP, KYN, and QUNA in supernatant of HULEC-5a cells treated with vehicle or IFNγ (10 ng/mL) for 48 h are shown. **D** Relative mRNA expression levels of major metabolic enzymes of KYN pathway (*KMO*, *KYNU*, and *3-HAO*) in differentiated macrophage-like cell lines (dTHP1 and dU937) and HULEC-5a cells. Each bar represents the mean ± standard deviation of three independent experiments. Statistical significance of mean values was tested using Student’s t-test (ns, not significant; **p-value < 0.01; ***p-value < 0.001). Bonferroni correction was conducted for multiple comparisons

In addition to in macrophages, the basal protein expression of IDO1 occurs in ECs in normal human lung tissues [37]. Additionally, predominant IDO1 expression can be observed in lung ECs of COVID-19 patients [38, 39], in whom elevated serum KYN and QUNA concentrations have been reported [40]. To clarify whether lung ECs contribute to the production of KYN and QUNA upon changes in immunological conditions, we investigated the effect of various inflammatory and anti-inflammatory stimuli on *IDO1* mRNA expression using immortalized normal human lung microvascular ECs (HULEC-5a cells) (Additional file 3: Fig. S8C). The significant induction of *IDO1* mRNA expression was observed upon stimulation with IFNβ, IFNγ, and TNFα (adjusted *p*-value < 0.01). Among these cytokines, IFNγ was the most potent inducer of *IDO1* mRNA expression; an over 20,000-fold increase in *IDO1* mRNA levels was observed after its application (Fig. 5A and Additional file 3: Fig. S7C). However, a decrease in mRNA levels upon anti-inflammatory stimulation was not observed.

The protein expression of IDO1 was analyzed using HULEC-5a cells treated with IFNγ. Western blotting showed no basal IDO1 protein expression in control HULEC-5a cells but increased protein expression levels after IFNγ treatment (Fig. 5B). Furthermore, we examined the effects of IFNγ treatment on the concentrations of TRP, KYN, and QUNA in the supernatant of HULEC-5a cells (Fig. 5C). TRP levels were significantly decreased to 3.9%, whereas KYN levels were increased (34.1-fold) by IFNγ treatment (*p-*value < 0.0001, Student’s t-test, Fig. 5C). However, contrary to the results for differentiated macrophage-like cell lines, QUNA levels in the cell supernatant of HULEC-5a were not affected by the treatments (Fig. 5C).

To clarify the reasons for the unchanged levels of QUNA in HULEC-5a cells, we analyzed the mRNA expression of metabolic enzymes involved in QUNA generation from KYN, such as KMO, KYNU, and 3-HAO (Fig. 2A). Interestingly, the mRNA levels of these enzymes in HULEC-5a cells were not detected or were much lower than those in dU937 and dTHP1 cells (adjusted *p-*values < 0.001, Student’s t-test with Bonferroni correction, Fig. 5D). These findings suggest that the metabolic pathway from KYN to QUNA is not active in HULEC-5a cells. As such, lung ECs in patients with acute DILD may facilitate rapid KYN generation and secretion upon IFNγ stimulation via the induction of IDO1 expression, but they are not the key cells in QUNA production in the lung.

### The induction of IFNγ levels in serum of DILD patients

Finally, to examine the association of IFNγ with the increase of KYN and QUNA in the serum of acute DILD patients who presented with high KYN and QUNA levels in serum, we compared serum IFNγ levels in samples obtained from patients with acute DILD, their matched-pair samples in the DILD recovery phase, and HC (n = 21, 14, 18). As expected, the IFNγ levels in all-DILD recovery patients and HC were below the detection limit (0.469 pg/mL), whereas IFNγ levels in some of the acute DILD patients were significantly high (Additional file 3; Fig. S10). Taken together, these findings support our data from in vitro experiments and indicate that upregulated IFNγ signaling has potentials to contribute to the increase in KYN and QUNA levels in the serum of patients with acute DILD.

## Discussion

In the current study, metabolomic analysis of serum samples was used to screen for novel DILD biomarkers. KYN levels were higher in patients with acute DILD than in recovered patients, which was consistent with a previous metabolomic study using serum from a limited number of patients with DILD and rheumatoid arthritis [41]. Moreover, quantitative analyses showed that the serum levels of KYN and the downstream metabolite, QUNA, and KYN/TRP ratios were higher in patients with acute DILD than in those in the recovery phase, as well as in HC and DILD-tolerant patients. Our data pave the way toward the future clinical application of these metabolites and the concentration ratio as new DILD biomarkers for the diagnosis of DILD onset and its recovery.

When diagnosing DILD, it is important to rule out the possibility of pneumonia attributed to infection and underlying diseases. In this regard, the clinically available serum biomarkers (KL-6 and SP-D) for ILDs have limitations in their lack of ability to distinguish between DILD and IIPs or CTD [10, 13–15]. On the contrary, the novel DILD biomarkers found in this study exhibited excellent diagnostic performance in discriminating DILD from IIPs and CTD, thereby overcoming the limitations of conventional biomarkers. Therefore, serum KYN and QUNA levels and the KYN/TRP ratio can serve as DILD-specific biomarkers for the differential diagnosis of DILD, which would contribute to clinical decisions regarding the discontinuation of suspected drugs being administered.

Similar to the biomarkers identified in this study, we have reported that plasma LPC(14:0) levels can be used as a biomarker for discriminating DILD from IIPs and CTD [19]. However, the drawbacks of LPC(14:0) in the differential diagnosis of DILD include that its plasma concentration also changes in patients with BP. In this regard, KYN-related biomarkers are more DILD-specific as significant differences in biomarker levels between DILD and BP were observed. Nevertheless, the combination of KYN-related biomarkers and LPC(14:0), along with conventional ILD biomarkers, might increase specificity for DILD diagnosis, which needs to be examined in future studies.

Before establishing KYN and QUNA as DILD biomarkers, it is important to elucidate why KYN and QUNA concentrations are altered in DILD patients. In humans, KYN is generated from TRP and converted into QUNA via the KYN pathway. Increased serum KYN and QUNA levels were observed in parallel with decreased serum TRP levels, thus TRP metabolism via the KYN pathway is likely activated in acute DILD patients, which is supported by the finding that the KYN/TRP ratio can serve as a biomarker. As macrophages are present in lesions of ILD [42] and recruitment of blood monocytes followed by their differentiation into macrophages is important in regulating lung inflammation [43], we first analyzed the effect of macrophage differentiation of monocytic THP1 and U937 cells on activation of the KYN pathway. Our data demonstrated that the induction of *IDO1* mRNA expression and elevated levels of KYN pathway metabolites in the extracellular space corresponded with cell differentiation. These findings suggest that differentiation of infiltrated monocytes into macrophages might be related to IDO1-mediated activation of the KYN pathway in the lung tissues of patients with acute DILD.

We next investigated the effect of various immune stimuli on KYN pathway activation in differentiated macrophages and lung ECs, finding that IDO1 expression is mainly induced by inflammatory cytokines. IFNγ is the most potent inducer of its expression in both macrophage-like and ECs. IFNγ-mediated activation of the KYN pathway was observed at both the protein and metabolite levels. These findings are consistent with earlier reports showing that IDO1 has a promoter region harboring several IFNγ-stimulated response elements and gamma activation sequences, and can be a transcriptional target gene of IFNγ-mediated intracellular signals [44–46]. In addition to the in vitro data, our results demonstrated that elevated IFNγ levels could be found only in acute DILD patients, but not in recovery patients and HC. Taken together, our results suggest that enhanced IDO1 expression in macrophage cells and lung ECs triggered by immune stimuli, as exemplified by IFNγ, may be a potential mechanism for the altered KYN concentration in the systemic circulation of acute DILD patients.

Contrary to our expectations, IFNγ-mediated induction of QUNA levels was only observed in macrophage-like cells but not in ECs. Further expression analyses of metabolic enzymes downstream of IDO1 revealed that the expression levels of metabolic enzymes required to produce QUNA from KYN in ECs were strikingly lower than those in macrophage-like cells. We infer that macrophages, but not ECs, play important roles in QUNA production in patients with DILD. However, since we only used ECs and macrophage-like cells here, QUNA may also be generated by other cell types in the lungs of patients with acute DILD, which needs to be examined in future studies.

The biological functions of KYN and QUNA under pathological conditions may inform their roles in DILD. A direct association between KYN metabolites and DILD is lacking, but many studies have demonstrated the role of the KYN pathway in immunosuppression [47]. For instance, starvation and metabolism of TRP in the microenvironment suppress the mTOR pathway and activate general control non-depressible 2 kinase, leading to cell cycle arrest and anergy of T cell infiltration, and induction of regulatory T (Treg) cells [48–51]. Further, KYN is an endogenous ligand for aryl hydrocarbon receptor (AhR) which induces FoxP3 (Treg marker) associated transcripts and the anti-inflammatory cytokine IL-10, resulting in production of Treg populations [52–55]. These findings, along with our results, imply that reduction of TRP and increase of KYN in differentiated macrophages and lung ECs may trigger immunosuppression in the lungs of acute DILD patients to alleviate excessive inflammation. The recent finding that IDO-AhR-mediated immunosuppression is critical for inhibiting acute lethal pulmonary inflammation caused by allogeneic hematopoietic stem cell transplantation [33] supports this hypothesis.

QUNA is classically known as a bioactive metabolite capable of activating the *N*-methyl-d-aspartate (NMDA) receptor [56, 57]. Additionally, it has been reported as a substrate for de novo synthesis of nicotinamide adenine dinucleotide (NAD) [58]. Given that increased levels of NAD and inhibition of NMDA receptors attenuate bleomycin-induced acute lung injury [59–61], we speculate that QUNA is not just a terminal metabolite of KYN destined for urinary excretion but may have biological roles in patients with acute DILD. However, the role of QUNA in the development of DILD remains unclear. Further studies focusing on the pathophysiological roles of KYN pathway metabolites in patients with DILD are necessary, which might lead to new therapeutic strategies for DILD.

Finally, before using serum KYN, QUNA, and the KYN/TRP ratio as DILD biomarkers in clinical settings, several caveats must be considered. First, food consumption before blood sampling was not completely controlled in the patients recruited for this study, and we recommend future tests on the effects of fasting prior to blood sampling on the concentration of biomarkers. Secondly, although it was shown that KYN and QUNA levels were significantly higher in acute DILD patients than the various related lung diseases that were tested, and that their levels were not notably affected by underlying diseases, to further analyze the specificity of these biomarkers, their serum levels in other inflammatory diseases and viral diseases, in which IFN signaling pathways are activated, should be examined. Third, as IFNα and IFNβ have been shown to induce *IDO1* mRNA expression, serum KYN and QUNA levels might not be suitable as DILD biomarkers in patients receiving IFN therapies. Fourth, since we analyzed the diagnostic potential of novel DILD biomarkers and determined their cutoff values retrospectively in the current study, further validation of the cutoff values, along with the clinical utility of KYN, QUNA, and KYN/TRP in prospective studies, is strongly warranted. Evaluating the clinical usefulness of the combination of current biomarkers with conventional biomarkers for DILD diagnosis awaits future studies. Lastly, non-invasive application of these biomarkers for DILD patients would minimize pain and stress during specimen collection. In the future, as our previous metabolomic study has shown that serum QUNA is concentrated approximately 100-fold in the urine of healthy donors [23], it will be interesting to examine the potential of urinary QUNA as a non-invasive DILD biomarker.

## Conclusions

Serum concentrations of KYN and QUNA and KYN/TRP ratio are promising biomarkers for detecting and monitoring DILD and its recovery. Combined with definitive diagnosis by HRCT, these markers could facilitate accurate decisions for the appropriate clinical management of patients with DILD. Moreover, the new biomarkers demonstrated a higher diagnostic potential than conventional ILD biomarkers (KL-6 and SP-D) when discriminating DILD from IIPs and CTD. Therefore, the use of the new biomarkers would aid not only in detecting DILD specifically but also making decisions regarding the continued use of medications in patients with IIPs and CTD who are suspected to have DILD based on the results of conventional biomarkers. In addition to the biomarkers' diagnostic potential, we also demonstrated a potential association between DILD and activation of the KYN pathway through inflammatory stimulation, as typified by IFNγ. Further studies are required to understand their biological role in DILD.

### Supplementary Information

**Additional file 1: Table S1.** Raw data on biomarker concentrations and baseline characteristics of each DILD and DILD recovery sample. # Some samples could not be masured due to insufficient serum available for the quantitative analysis. **Table S2.** Raw data on biomarker concentrations and baseline characteristics of each serum sample other than DILD. **Table S3.** Metabolite peak areas measured by HILIC/TOF–MS in DILD serum samples. **Table S4.** PCR primer pairs used in this study. **Table S5.** Comparison of serum concentrations of conventional biomarkers among patient groups. Statistical differences between the two groups were examined using the Mann–Whitney U-test with Bonferroni correction. The adjusted *p*-values are presented in the table. The patients with missing values of the conventional biomarkers were excluded from the statistical analyses. DILD recovery: patients recovered from DILD, DILD-tolerant: the patient group taking similar medications to the DILD group but without DILD onset, BP: bacterial pneumonia, NTM: nontuberculous mycobacteriosis, IIPs: idiopathic interstitial pneumonias, CTD: lung disease associated with connective tissue disease, COPD: chronic obstructive pulmonary disease, BA: bronchial asthma, N.S.: not significant. **Table S6.** Positive rate of the identified DILD biomarker candidates and conventional biomarkers. The optimal cutoff values of KYN, QUNA, and KYN/TRP were determined using Youden’s index of the ROC curve built between the all-DILD and DILD recovery groups. The positivity rates for other conventional biomarkers were calculated using clinically used cutoff values. The cutoff values for biomarkers are indicated in the parenthesis. **Table S7.** DILD biomarker potential of tryptophan metabolites compared to serum biomarkers in OP and NSIP patients. AUROC values were calculated from ROC curves, and those with statistical significance (*p*-value < 0.05) are indicated in bold letters. DILD recovery: patients recovered from DILD, DILD-tolerant: the patient group taking similar medications to the DILD group but without DILD onset, BP: bacterial pneumonia, NTM: nontuberculous mycobacteriosis, IIPs: idiopathic interstitial pneumonias, CTD: lung disease associated with connective tissue disease, COPD: chronic obstructive pulmonary disease, BA: bronchial asthma, HC: healthy control, N.A.: not applicable.

**Additional file 2. **Supplemental Methods. Sample extraction methods and analytical methods for the measurement of TRP, KYN, and QUNA are described in detail.

**Additional file 3: Figure S1.** Comparisons of KYN concentrations between DILD and recovery patients in a validation cohort. The serum concentration of KYN in DILD (n = 22) and recovery (n = 17) patients in a validation cohort are shown in box-and-whisker plots. The statistical significance between the DILD and recovery groups was tested using the Mann–Whitney U-test. *; *p*-value < 0.05. **Figure S2.** Distributions of TRP concentrations in DILD, other lung diseases, and HC. Serum TRP concentrations among the sample groups are shown in the box-and-whisker plot. The results of the statistical comparisons among the groups are summarized in Table 2. DAD/DAD-mixed: DILD patients in acute phase with CT pattern of diffuse alveolar damage, OP: DILD patients in acute phase with CT pattern of organizing pneumonia, NISP: DILD patients in acute phase with CT pattern of nonspecific interstitial pneumonia: Other: DILD patients in acute phase with CT pattern other than DAD, OP and NSIP, DILD recovery: patients recovered from DILD, DILD-tolerant: the patient group taking similar medications to the DILD group but without DILD onset, BP: bacterial pneumonia, NTM: nontuberculous mycobacteriosis, IIPs: idiopathic interstitial pneumonias, CTD: lung disease associated with connective tissue disease, COPD: chronic obstructive pulmonary disease, BA: bronchial asthma, HC: healthy control. **Figure S3.** Sensitivity analysis of the sample selection bias. To evaluate sample selection bias, the samples were divided into two subcohorts based on the location of the hospitals (Chiba University and Nippon Medical School; Tokyo metropolitan area [sub-cohort A], Shinshu University and Hiroshima University; and the other area [sub-cohort B], Additional file 1: Table S1). Sub-cohort A included 41 DILD patients and 28 recovery patients, whereas sub-cohort B included 40 DILD patients and 25 recovery patients. **A** KYN, **B** QUNA), **C** TRP concentrations, **D** KYN/TRP ratio in each sub-cohort are shown in box-and-whisker plots. The statistical significance between the DILD and recovery groups in each sub-cohort was tested using the Mann–Whitney U-test. *; *p*-value < 0.05, **; *p*-value < 0.01, ***;* p*-value < 0.001, ****; *p*-value < 0.0001. Samples with missing values were excluded from the statistical analysis. **Figure S4.** Comparisons of the identified biomarker levels by underlying disease and type of medication for DILD. **A** The association of the existence of cancers with the serum concentrations of KYN and qQUNA, and KYN/TRP ratio in all-DILD patients was analyzed. DILD patients were divided into three groups based on the existence of cancers as underlying diseases. The metabolites concentrations in each group are shown as a box-and-whisker plot. The statistical significances were examined by Mann–Whitney U-test with Bonferroni correction. **B** Evaluation of the effect of the existence of lifestyle-related diseases on the serum concentrations of KYN and QUNA, and KYN/TRP ratio in all-DILD patients were analyzed. DILD patients were divided into subgroups based on the existence of lifestyle-related diseases (cardiovascular diseases, dyslipidemia, hypertension and diabetes). In case one patient have two different lifestyle-related diseases, the data were assigned to both groups. The metabolite concentrations in each group are shown as a box-and-whisker plot. The statistical significances were tested by Mann–Whitney U-test with Bonferroni correction. **C** The association of types of medications with the serum concentrations of KYN and QUNA, and KYN/TRP ratio in all-DILD patients was analyzed. DILD patients were divided into subgroups based on types of medications. If a patient had two different types of medications, the data were assigned to both groups. The metabolite concentrations in each group are shown as a box-and-whisker plot. The statistical significances were tested by Mann–Whitney U-test with Bonferroni correction. DDAs: DNA damaging agents, ICIs: immune checkpoint inhibitors, TKIs: tyrosine kinase inhibitors. **Figure S5.** Correlation of KYN/TRP ratio with severity and mortality of DILD. **A** Correlation analysis of KYN/TRP ratio with SpO_2_/FiO_2_ ratio in acute phase DILD patients (n = 44). **B** Comparison of serum KYN/TRP ratio between DILD patients who survived and those who died due to DILD. The DILD patients who died from causes unrelated to DILD were excluded from the analysis. The statistical significances were tested by Mann–Whitney U-test. ns; not significant. **Figure S6.** Distribution of serum concentrations of conventional biomarkers in DILD and other lung diseases. Serum concentrations of SP-D **A**, KL-6 **B** and CRP **C** were measured in serum samples obtained from patients with DILD, DILD-tolerant patients, and patients with other lung diseases. Biomarker levels are shown in box-and-whisker plots. The number of samples in each group is summarized in Additional file 1: Table S1 and S2, and the results of the statistical comparisons among the groups are summarized in Table S5. DAD/DAD-mixed: DILD patients in acute phase with CT pattern of diffuse alveolar damage, OP: DILD patients in acute phase with CT pattern of organizing pneumonia, NISP: DILD patients in acute phase with CT pattern of nonspecific interstitial pneumonia: Other: DILD patients in acute phase with CT pattern other than DAD, OP and NSIP, DILD recovery: patients recovered from DILD, DILD-tolerant: the patient group taking similar medications to the DILD group but without DILD onset, BP: bacterial pneumonia, NTM: nontuberculous mycobacteriosis, IIPs: idiopathic interstitial pneumonias, CTD: lung disease associated with connective tissue disease, COPD: chronic obstructive pulmonary disease, BA: bronchial asthma. **Figure S7.** Diagnostic potentials of KYN, QUNA, KYN/TRP ratio, and conventional DILD biomarkers in DAD/DAD-mixed patients. ROC curve analyses of serum levels of KYN and QUNA, KYN/TRP ratio, and levels of conventional ILD biomarkers (SP-D and KL-6) were performed between the groups using the quantitative data in the combined cohort. The ROC curves of DAD/DAD-mixed patients compared with DILD-tolerant **A,** DILD recovery **B**, IIPs **C**, or CTD **D** are shown. The values of AUROC are described in the parentheses of the labels for each tested biomarker. The AUROC values for other comparisons are summarized in Table 3. **Figure S8.** Effect of various inflammatory and anti-inflammatory stimuli on *IDO1* mRNA expression in differentiated macrophage cell lines and a lung endothelial cell line. Fold changes of *IDO1* mRNA expression levels upon various inflammatory and anti-inflammatory stimuli (10 ng/mL for all cytokines) were examined in differentiated macrophage cell lines (**A** dTHP1 and **B** dU937) and lung ECs (**C** HULEC-5a). The error bar represents the mean ± standard deviation of three independent experiments. Statistical significance of *IDO1* mRNA levels between control cells and cytokine-treated cells was tested using Student’s t-test with Bonferroni correction (ns, not significant; **, adjusted *p*-value < 0.01; ***, adjusted *p*-value < 0.001). **Figure S9.** Induction of KYN pathway metabolites in cell lysates and supernatant of differentiated U937 cells treated with IFNγ. Relative levels of TRP, KYN, and QUNA in supernatant **A** and whole cell lysate **B** of dU937 cells treated with 10% FBS-PBS (control) or IFNγ (10 ng/mL) for 24 h are shown. Each bar represents the mean ± standard deviation of three independent experiments. Statistical significance of mean values was tested using Student’s t-test (ns, not significant; **, *p-*value < 0.01; ***, *p-*value < 0.001). **Figure S10.** Serum IFNγ levels in DILD, DILD recovery, and HC. Serum IFNγ levels in patients with DILD showing high serum KYN and QUNA levels (n = 21), matched pair recovery samples (n = 14), and HC (n = 18) were measured using a commercially available sandwich ELISA kit. The matched-pair samples between the DILD and DILD recovery groups are indicated by solid lines. The limit of detection (LOD) was 0.469 pg/mL. It has been demonstrated that some patients with DILD showed detectable and elevated serum IFNγ levels, while their levels in all-DILD recovery patients and HC were below the detection limit.

**Additional file 4. **Uncropped images of Western blotting analysis for IDO1 and GAPDH in dU937 and dTHP1 cells.

**Additional file 5. **Uncropped images of Western blotting analysis for IDO1 and GAPDH in HULEC-5a cells.

## Data Availability

The raw data of metabolomic and quantitative analysis are available in the supplementary information files. The other analyzed data in the current study are available from the corresponding author on reasonable request.

## Abbreviations

ACN: Acetonitrile
AUROC: Area under the ROC curve
AhR: Aryl hydrocarbon receptor
BP: Bacterial pneumonia
BSA/TBS-T: Bovine serum albumin in Tris-buffered saline with 0.05% Tween 20
BA: Bronchial asthma
COPD: Chronic obstructive pulmonary disease
CRP: C-reactive protein
dTHP1: Differentiated THP1
dU937: Differentiated U937
DAD: Diffuse alveolar damage
DILD: Drug-induced interstitial lung disease
FBS: Fetal bovine serum
GAPDH: Glyceraldehyde-3-phosphate dehydrogenase
HC: Healthy controls
HRCT: High-resolution computed tomography
HILIC/TOF–MS: Hydrophilic interaction liquid chromatography coupled to time-of-flight mass spectrometry
IIPs: Idiopathic interstitial pneumonias
IDO1: Indoleamine 2,3-dioxygenase 1
IFN: Interferon
IL: Interleukin
ILD: Interstitial lung disease
IS: Internal standards
IC/MS: Ion chromatography coupled to high-resolution Orbitrap mass spectrometry
KL-6: Krebs von den Lungen-6
KYN: Kynurenine
CTD: Lung disease associated with connective tissue disease
MRM: Multiple-reaction monitoring
NAD: Nicotinamide adenine dinucleotide
NMDA: *N*-Methyl-d-aspartate
NSIP: Nonspecific interstitial pneumonia
NTM: Nontuberculous mycobacteriosis
ROC: Operating characteristic curve
RPMI-1640: Roswell Park Memorial Institute medium 1640
OP: Organizing pneumonia
PMA: Phorbol 12-myristate 13-acetate
PBS: Phosphate-buffered saline
QUNA: Quinolinic acid
Treg: Regulatory T
RT-qPCR: Reverse transcription-quantitative real-time PCR
RP-LC/MS: Reverse-phase liquid chromatography coupled to a triple quadrupole mass spectrometry
SP-D: Surfactant protein-D
TRP: Tryptophan
TNFα: Tumor necrosis factor α
