# Supplementary material for: Identification of kynurenine and quinolinic acid as promising serum biomarkers for drug-induced interstitial lung diseases
Source: Respir Res. 2024 Jan 14;25:31. doi: 10.1186/s12931-023-02653-6 (PMC10788992; doi:10.1186/s12931-023-02653-6)
Supplement: Supplementary file 2 — Additional file 2. Supplemental Methods. Sample extraction methods and analytical methods for the measurement of TRP, KYN, and QUNA are described in detail. [file 12931_2023_2653_MOESM2_ESM.docx]

**Supplemental Methods**

**Preparation of calibration standards for quantitative analysis of tryptophan, kynurenine, and quinolinic acid**

Calibration standards were prepared in water at concentrations of 0.10, 0.19, 0.48, 0.96, 1.92, 4.80, 9.61, and 19.21 µM for kynurenine (KYN) and 0.73, 1.47, 3.67, 7.34, 14.69, 36.72, 73.45, and 146.89 µM for tryptophan (TRP). We chose quinolinic acid d3 (QUNA-d3) as a calibration standard molecule for QUNA quantification as a matrix effect on the detection of QUNA was observed in serum samples that existed even in serum from healthy subjects. The ionization efficiencies of QUNA and QUNA-d3 at the same concentration were comparable (data not shown). Calibration standards for QUNA were prepared in commercially available normal human pooled serum at concentrations of 20, 50, 100, 400, 1000, 2000, and 5000 nM QUNA-d3.

**Sample extraction for quantitative analyses of KYN pathway metabolites in serum samples**

The sample preparation methods for TRP/KYN and QUNA were different because they were separately quantified using different analytical platforms, the methodologies for which are as follows.

To extract TRP and KYN from serum samples, 30 µL of serum or calibration standards was mixed with 470 µL of 85% methanol in water containing the internal standard (IS) (40 nM KYN-d6 [L-KYN sulfate (ring-D4,3,3-D2)] and 478 nM TRP-d5) and centrifuged (18,440 or 21,210 × *g*, 10 min, 4°C) to remove proteins. The solution was transferred to a FastRemoverC18 (GL Science, Tokyo, Japan) using Microlab NIMBUS with an MPE2 unit (Hamilton, Reno, NV). The collected flow-through fraction was diluted with three times the volume of water and subjected to quantitative analysis. For samples from healthy controls, the preparation method was slightly modified. Human serum (50 µL) was mixed with 450 µL of 90% methanol in water containing IS and vortexed. The mixture was filtered using Fast Remover for Protein (GL Science). The filtered samples were submitted to FastRemoverC18 in the automated system and the flow-through fraction was diluted as described above.

The QUNA extraction methods were optimized using the extraction method established in a previous study [1]. QUNA-d3 was used as a calibration standard and hippuric acid-d5 was chosen as the IS for QUNA quantification because the retention time is close to that of QUNA and QUNA-d3 in the ion chromatography coupled to high-resolution Orbitrap mass spectrometry (IC/MS)-based quantification method. To extract QUNA, 20 µL of serum or calibration standards was mixed with 80 µL of water and 400 µL of methanol containing IS (100 nM hippuric acid-d5). Deproteinization and delipidation were conducted using a previously described method [1]. After evaporation, the extracted samples were dissolved in 80 µL of water and filtered using an Ultrafree-MC GV Centrifugal Filter (Merck Millipore, Burlington, MA).

**Quantification of serum concentrations of KYN pathway metabolites**

In the quantitative analyses, TRP and KYN levels were measured using a reverse-phase liquid chromatography coupled to a triple quadrupole mass spectrometry (RP-LC/MS) system, whereas QUNA level was measured using the IC/MS system in targeted approaches. Both quantification methods were appropriately validated with reference to the points to consider in the document on analytical assay validation for biomarkers [2] and guidelines/guidance on analytical assay validation for drugs [3-5], and all validated results met the acceptance criteria of the guidelines/guidance.

For TRP and KYN measurements, the prepared samples were separated by LC using an Ultimate 3000 system (Thermo Fisher Scientific, Waltham, MA) with a Triart PFP column (1.9 μm, 2.1 × 100 mm; YMC, Kyoto, Japan) at 45°C. Mobile phase A consisted of water with 0.3% formic acid and mobile phase B consisted of methanol with 0.3% formic acid. The volume of the injected sample was 5 μL. After holding 20% B for 0.5 min at a flow rate of 0.3 mL/min, a ramp gradient was used as follows: 20% B at 0.5 min, increased to 100% B at 2.5 min, and held for 1.5 min before returning to 20% B at 4.1 min, which was held for 0.9 min. For mass detection, TSQ-Quantiva (Thermo Fisher Scientific) was operated in multiple-reaction monitoring (MRM) mode. Sample ionization was performed by heated electrospray ionization (ESI) in positive-mode. Ion source parameters were as follows: ion source voltage, 3.5 kV; ion-transfer tube temperature, 350°C; and vaporizer temperature, 250°C. The gas settings were as follows: sheath gas, 40 arbitrary units (Arbs); auxiliary gas, 10 Arbs; and sweep gas, 1 Arb. The following MRM transitions (m/z) were monitored at collision energies of 10 V with Ar 1.5: TRP 205.1/188.1, TRP-d5 210.1/192.0, KYN 209.1/192.1, and KYN-d6 215.1/198.1.

QUNA in the extracted samples was separated using Dionex ICS-5000+ (Thermo Fisher Scientific) with an IonPac AS11-HC-4 μm IC column (2 × 250 mm, Thermo Fisher Scientific). The potassium hydroxide gradient for sample separation was as follows: 10 mM to 100 mM, 0–7.0 min; 100 mM, 7.0–8.0 min; 100 mM to 10 mM, 8.0–8.1 min; and 10 mM, 8.1–10.1 min. The flow rate was set to 0.3 mL/min. An electrolytic suppressor was used to remove potassium ions in the post-column region to prevent their flow into the MS. To enhance ionization, a methanol line (flow rate: 0.1 mL/min) was merged with a transfer line from the IC to the MS. For the MS detection of QUNA, Q-Exactive (Thermo Fisher Scientific) was operated in heated ESI negative-mode. Target molecules were detected in parallel reaction monitoring (PRM) mode. The ESI source parameters were the same as those used in metabolomics [1]. The MS parameters for PRM measurements were as follows: resolution, 17,500; automatic gain control target, 2×10^5^; maximum injection time, 100 ms; isolation window, 1.0 m/z; fixed first mass, 50.0 m/z; and normalized collision energy (NCE), 15. The monitored MS transitions were as follows: QUNA, 166.0145/122.0251; QUNA-d3, 169.0334/125.0439; and hippuric acid-d5, 183.08233/183.08231. Because the NCE value used in the PRM mode was weak for the fragmentation of hippuric acid-d5, we monitored the accurate mass of precursor ions instead of product ions. The combination of QUNA and hippuric acid-d5 was used for the measurement of serum samples from patients and healthy controls, whereas the combination of QUNA-d3 and hippuric acid-d5 was used for the measurement of calibration standards.

Data were analyzed using TraceFinder 4.1 (Thermo Fisher Scientific). The calibration curves were constructed using the normalized peak area of each target molecule with a weighting factor of 1/X^2^ in GraphPad Prism 9 (GraphPad Software, San Diego, CA).

**Metabolite extraction from the cultured cells and their culture medium**

Metabolites were extracted from cell lysates of immune-stimulated macrophage-like cells. dTHP1 cells and dU937 cells that had undergone IFNγ stimulation were harvested and pelleted by centrifugation (200 × *g*, 5 min, 4°C). After washing with normal saline (HPLC grade), 1 × 10^6^ cells were pelleted again by centrifugation (800 × *g*, 5 min, 4°C) and resuspended in 100 µL of water. The samples were stored at -80°C until use.

The cell culture media of undifferentiated THP1 and U937 cells, differentiated macrophage-like cells (dTHP1 and dU937 cells), and HULEC-5a cells treated with IFNγ were collected. Cell debris was removed by centrifugation (10,000 × *g*, 1 min, 4°C), followed by filtration using a 0.22-µm PVDF membrane. The filtered samples (100 µL) were stored at -80°C until use.

To extract metabolites, samples (100 µL) were mixed with 400 µL of methanol containing IS (100 nM KYN-d6, 1000 nM TRP-d5, and 100 nM QUNA-d3). After vortexing for 1 min, the aggregated proteins were removed by centrifugation (15,000 × *g*, 2 min, 4°C), followed by filtration through a 0.22-µm PVDF membrane. Samples were delipidated using a MonoSpin C18 column (GL Science) equilibrated with methanol followed by water. The flow-through (250 µL) was evaporated and dissolved in 125 µL of water. The dissolved samples (50 µL) were mixed with 110 µL of water and 40 µL of methanol for the RP-LC/MS analysis of TRP and KYN. The remaining dissolved samples were filtered using a 0.22-µm PVDF membrane and subjected to IC/MS-based QUNA measurements. Measurements were conducted under the aforementioned analytical conditions. The peak areas of TRP, KYN, and QUNA were normalized to those of the corresponding IS molecules. The levels of each metabolite in the cell lysate and culture medium were calculated relative to the controls.

**References**

1. Sun Y, Saito K, Iiji R, Saito Y. Application of Ion Chromatography Coupled with Mass Spectrometry for Human Serum and Urine Metabolomics. SLAS Discov. 2019;24:778-86.
2. Biomarker Assay Collaborative Evidentiary Considerations Writing Group, Critical Path Institute. Points to Consider Document: Scientific and Regulatory Considerations for the Analytical Validation of Assays Used in the Qualification of Biomarkers in Biological Matrices. 2019. <https://c-path.org/wp-content/uploads/2019/06/evidconsid-whitepaper-analyticalsectionv2019.pdf> Accessed 21 July, 2023.
3. US FDA. Bioanalytical Method Validation Guidance for Industry. 2018. <https://www.fda.gov/files/drugs/published/Bioanalytical-Method-Validation-Guidance-for-Industry.pdf> Accessed 21 July, 2023.
4. EMA. Guideline on bioanalytical method validation. 2012. <https://www.ema.europa.eu/en/documents/scientific-guideline/guideline-bioanalytical-method-validation_en.pdf> Accessed 21 July, 2023.
5. Ministry of Health Labour and Welfare of Japan. Guideline on Bioanalytical Method Validation in Pharmaceutical Development. 2013. <https://www.pmda.go.jp/files/000206209.pdf> Accessed 21 July, 2023.
