# Supplementary material for: Identification of kynurenine and quinolinic acid as promising serum biomarkers for drug-induced interstitial lung diseases
Source: Respir Res. 2024 Jan 14;25:31. doi: 10.1186/s12931-023-02653-6 (PMC10788992; doi:10.1186/s12931-023-02653-6)
Supplement: Supplementary file 3 — Additional file 3: Figure S1. Comparisons of KYN concentrations between DILD and recovery patients in a validation cohort. The serum concentration of KYN in DILD (n = 22) and recovery (n = 17) patients in a validation cohort are shown in box-and-whisker plots. The statistical significance between the DILD and recovery groups was tested using the Mann–Whitney U-test. *; p-value < 0.05. Figure S2. Distributions of TRP concentrations in DILD, other lung diseases, and HC. Serum TRP concentrations among the sample groups are shown in the box-and-whisker plot. The results of the statistical comparisons among the groups are summarized in Table 2. DAD/DAD-mixed: DILD patients in acute phase with CT pattern of diffuse alveolar damage, OP: DILD patients in acute phase with CT pattern of organizing pneumonia, NISP: DILD patients in acute phase with CT pattern of nonspecific interstitial pneumonia: Other: DILD patients in acute phase with CT pattern other than DAD, OP and NSIP, DILD recovery: patients recovered from DILD, DILD-tolerant: the patient group taking similar medications to the DILD group but without DILD onset, BP: bacterial pneumonia, NTM: nontuberculous mycobacteriosis, IIPs: idiopathic interstitial pneumonias, CTD: lung disease associated with connective tissue disease, COPD: chronic obstructive pulmonary disease, BA: bronchial asthma, HC: healthy control. Figure S3. Sensitivity analysis of the sample selection bias. To evaluate sample selection bias, the samples were divided into two subcohorts based on the location of the hospitals (Chiba University and Nippon Medical School; Tokyo metropolitan area [sub-cohort A], Shinshu University and Hiroshima University; and the other area [sub-cohort B], Additional file 1: Table S1). Sub-cohort A included 41 DILD patients and 28 recovery patients, whereas sub-cohort B included 40 DILD patients and 25 recovery patients. A KYN, B QUNA), C TRP concentrations, D KYN/TRP ratio in each sub-cohort are shown in box-and-whisker plo [file 12931_2023_2653_MOESM3_ESM.pdf]

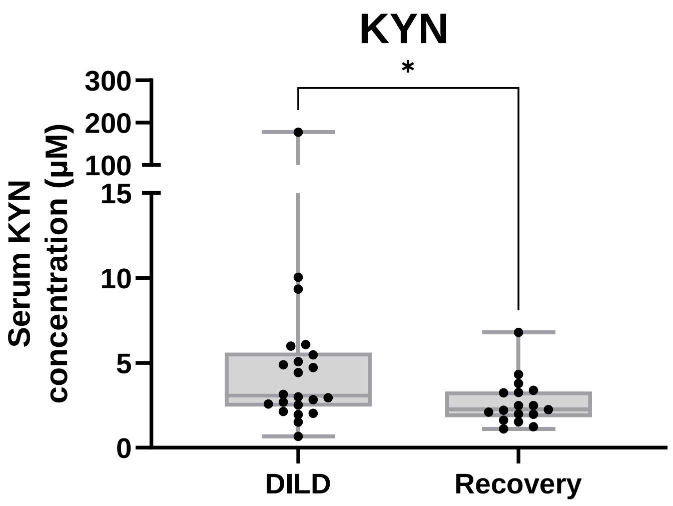

Figure S1. Comparisons of KYN concentrations between DILD and recovery patients in a validation cohort. The serum concentration of KYN in DILD (n=22) and recovery (n=17) patients in a validation cohort are shown in box-and-whisker plots. The statistical significance between the DILD and recovery groups was tested using the Mann–Whitney U-test. *; *p*-value < 0.05.

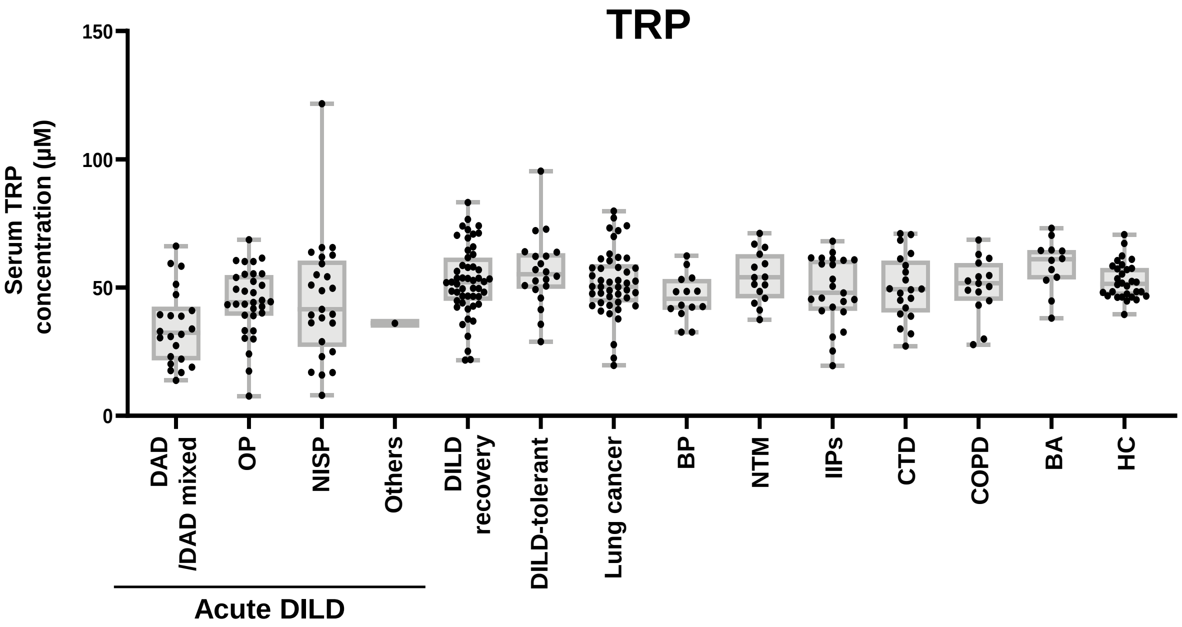

Figure S2. Distributions of TRP concentrations in DILD, other lung diseases, and HC. Serum TRP concentrations among the sample groups are shown in the box-and-whisker plot. The results of the statistical comparisons among the groups are summarized in Table 2. DAD/DAD-mixed: DILD patients in acute phase with CT pattern of diffuse alveolar damage, OP: DILD patients in acute phase with CT pattern of organizing pneumonia, NISP: DILD patients in acute phase with CT pattern of nonspecific interstitial pneumonia: Other: DILD patients in acute phase with CT pattern other than DAD, OP and NSIP, DILD recovery: patients recovered from DILD, DILD-tolerant: the patient group taking similar medications to the DILD group but without DILD onset, BP: bacterial pneumonia, NTM: nontuberculous mycobacteriosis, IIPs: idiopathic interstitial pneumonias, CTD: lung disease associated with connective tissue disease, COPD: chronic obstructive pulmonary disease, BA: bronchial asthma, HC: healthy control.

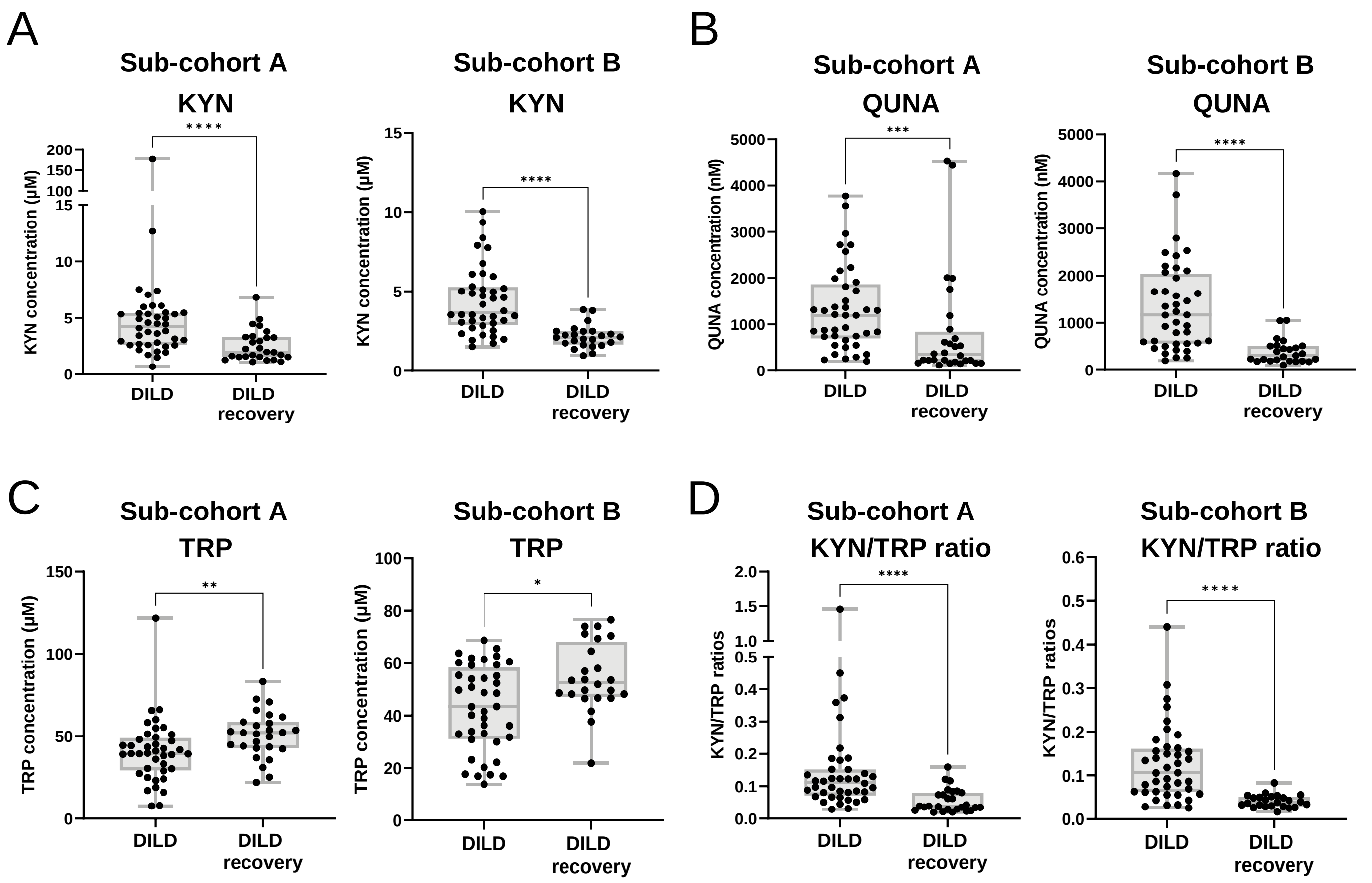

Figure S3. Sensitivity analysis of the sample selection bias.

To evaluate sample selection bias, the samples were divided into two subcohorts based on the location of the hospitals (Chiba University and Nippon Medical School; Tokyo metropolitan area [sub-cohort A], Shinshu University and Hiroshima University; and the other area [sub-cohort B], Additional file 1: Table S1). Sub-cohort A included 41 DILD patients and 28 recovery patients, whereas sub-cohort B included 40 DILD patients and 25 recovery patients. **A** KYN, **B** QUNA, **C** TRP concentrations, **D** KYN/TRP ratio in each sub-cohort are shown in box-and-whisker plots. The statistical significance between the DILD and recovery groups in each sub-cohort was tested using the Mann–Whitney U-test. *; *p*-value < 0.05, **; *p*-value < 0.01, ***; *p*-value < 0.001, ****; *p*-value < 0.0001. Samples with missing values were excluded from the statistical analyses.

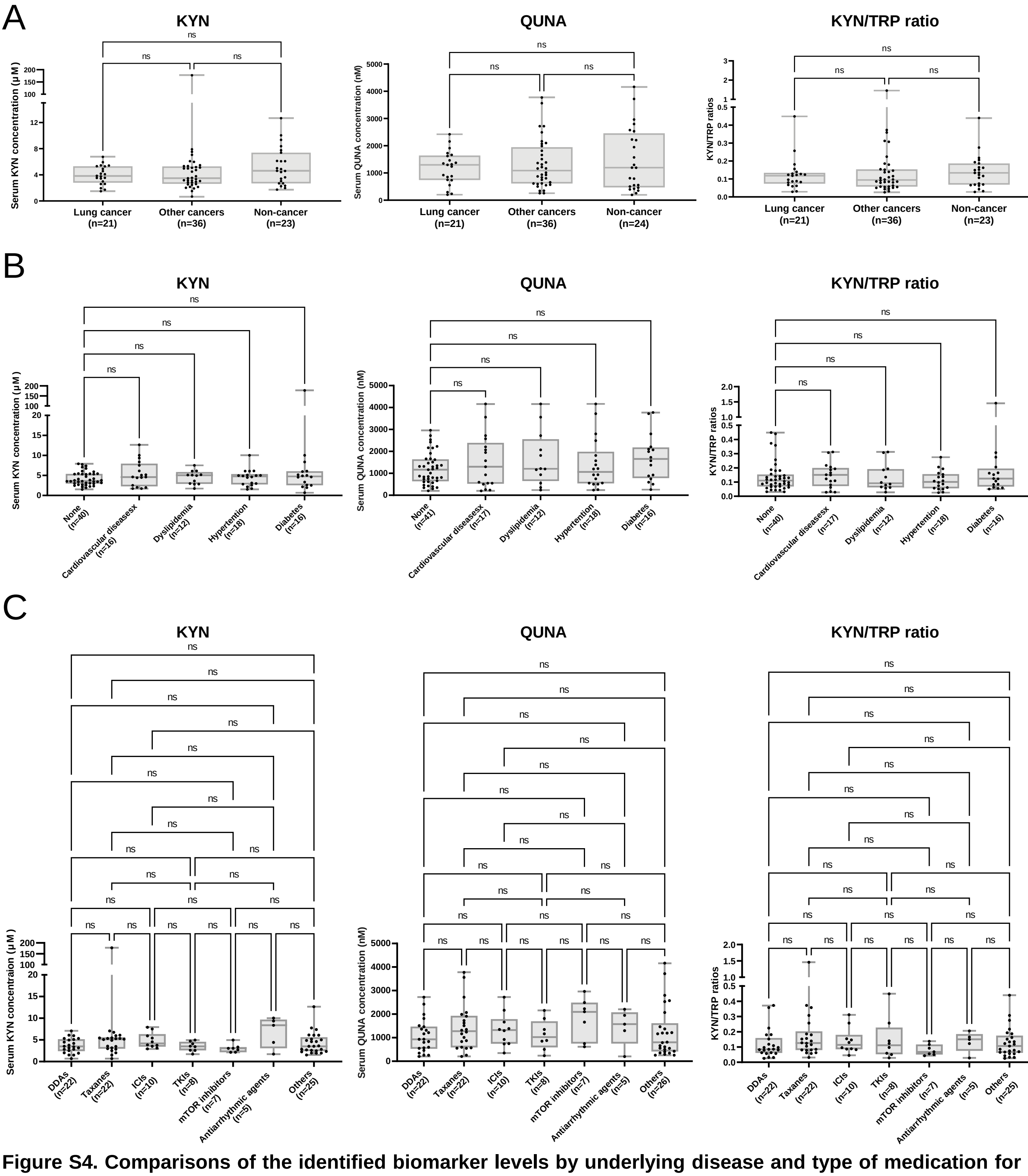

Figure S4. Comparisons of the identified biomarker levels by underlying disease and type of medication for DILD.

A The association of the existence of cancers with the serum concentrations of KYN and QUNA, and KYN/TRP ratio in all-DILD patients was analyzed. DILD patients were divided into three groups based on the existence of cancers as underlying diseases. The metabolites concentrations in each group are shown as a box-and-whisker plot. The statistical significances were examined by Mann-Whitney U-test with Bonferroni correction. **B** Evaluation of the effect of the existence of lifestyle-related diseases on the serum concentrations KYN and QUNA, and KYN/TRP ratio in all-DILD patients were analyzed. DILD patients were divided into subgroups based on the existence of lifestyle-related diseases (cardiovascular diseases, dyslipidemia, hypertension and diabetes). In case one patient have two different lifestyle-related diseases, the data were assigned to both groups. The metabolite concentrations in each group are shown as a box-and-whisker plot. The statistical significances were tested by Mann-Whitney U-test with Bonferroni correction. **C** The association of types of medications with the serum concentrations of KYN and QUNA, and KYN/TRP ratio in all-DILD patients was analyzed. DILD patients were divided into subgroups based on types of medications. If a patient had two different types of medications, the data were assigned to both groups. The metabolite concentrations in each group are shown as a box-and-whisker plot. The statistical significances were tested by Mann-Whitney U-test with Bonferroni correction. DDAs: DNA damaging agents, ICIs: immune checkpoint inhibitors, TKIs: tyrosine kinase inhibitors.

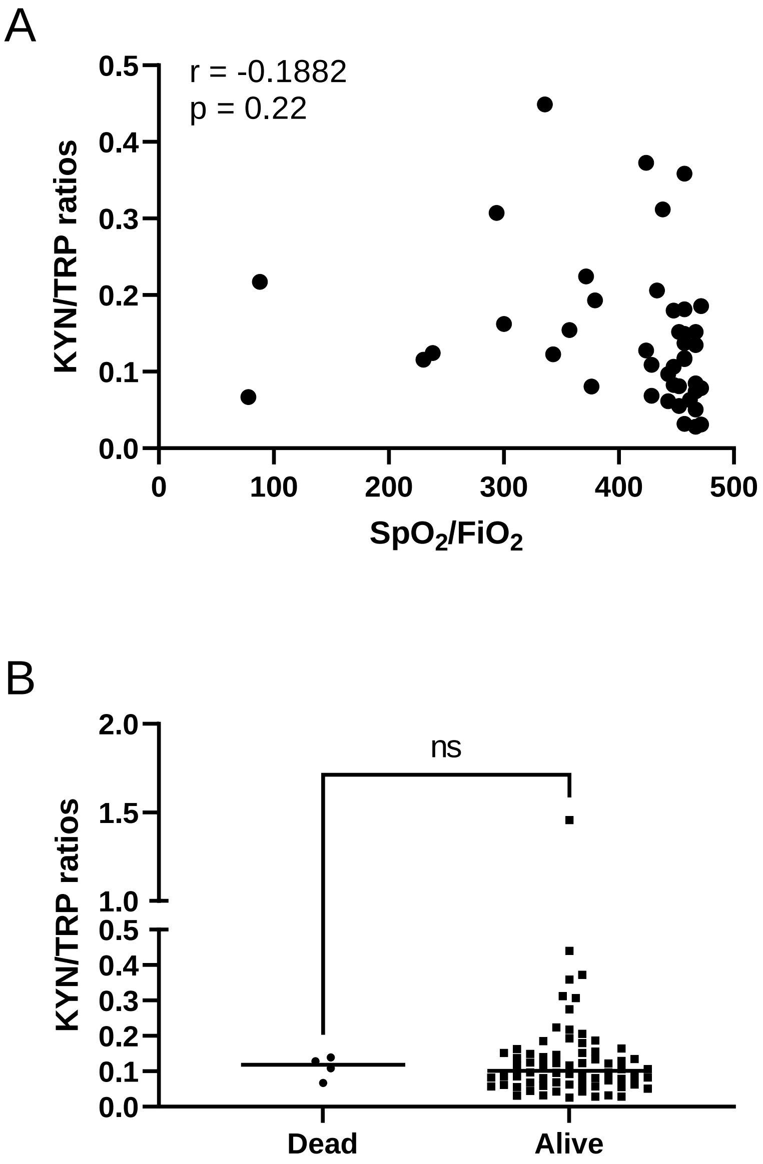

Figure S5. Correlation of KYN/TRP ratio with severity and mortality of DILD.
A Correlation analysis of KYN/TRP ratio with SpO_2/FiO_2 ratio in acute phase DILD patients (n=44). **B** Comparison of serum KYN/TRP ratio between DILD patients who survived and those who died due to DILD. The DILD patients who died from causes unrelated to DILD were excluded from the analysis. The statistical significances were tested by Mann-Whitney U-test. ns; not significant.

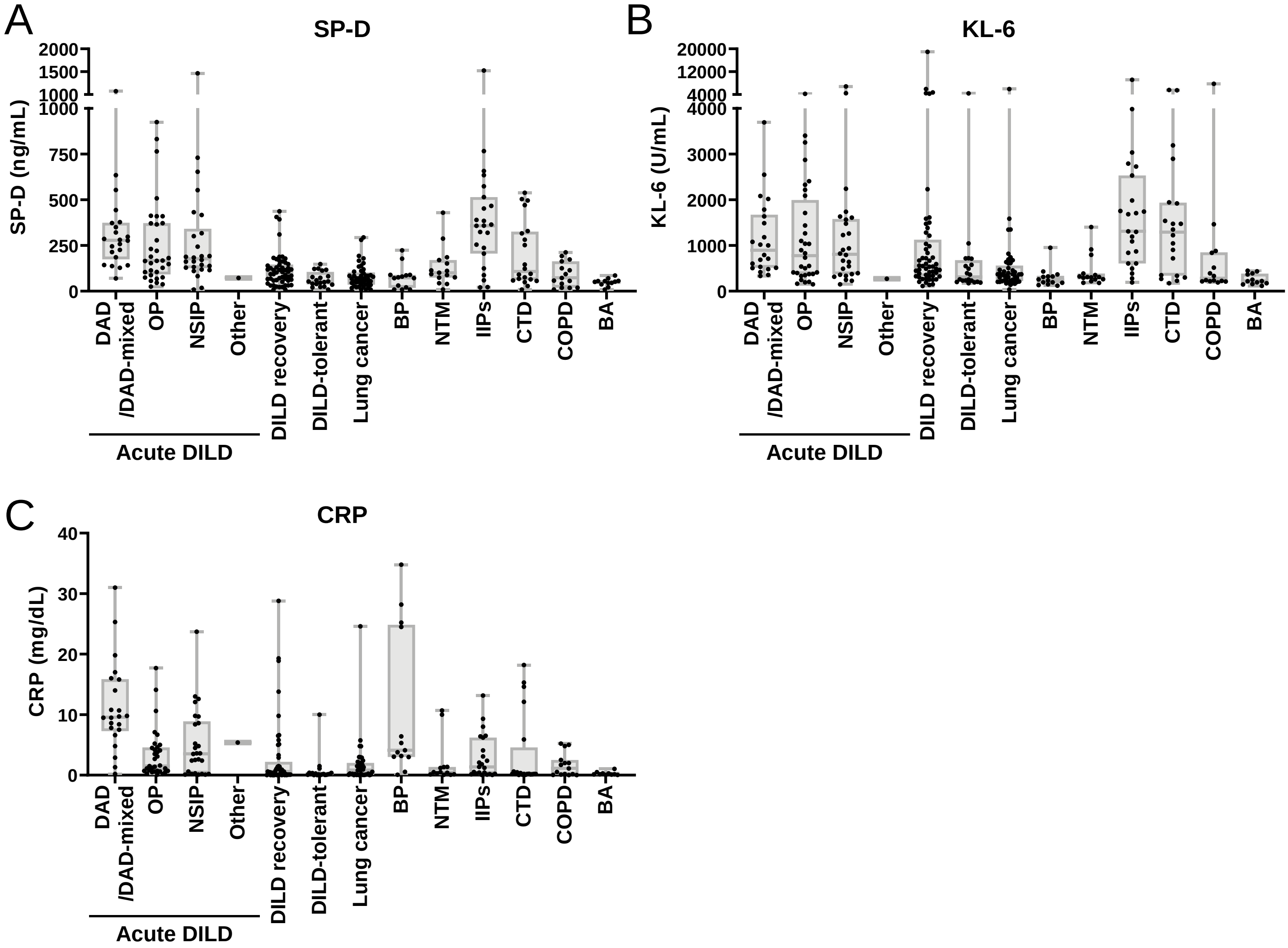

Figure S6. Distribution of serum concentrations of conventional biomarkers in DILD and other lung diseases. Serum concentrations of SP-D **A**, KL-6 **B** and CRP **C** were measured in serum samples obtained from patients with DILD, DILD-tolerant patients, and patients with other lung diseases. Biomarker levels are shown in box-and-whisker plots. The number of samples in each group is summarized in Additional file 1: Table S1 and S2, and the results of the statistical comparisons among the groups are summarized in Table S5. DAD/DAD-mixed: DILD patients in acute phase with CT pattern of diffuse alveolar damage, OP: DILD patients in acute phase with CT pattern of organizing pneumonia, NISP: DILD patients in acute phase with CT pattern of nonspecific interstitial pneumonia: Other: DILD patients in acute phase with CT pattern other than DAD, OP and NSIP, DILD recovery: patients recovered from DILD, DILD-tolerant: the patient group taking similar medications to the DILD group but without DILD onset, BP: bacterial pneumonia, NTM: nontuberculous mycobacteriosis, IIPs: idiopathic interstitial pneumonias, CTD: lung disease associated with connective tissue disease, COPD: chronic obstructive pulmonary disease, BA: bronchial asthma.

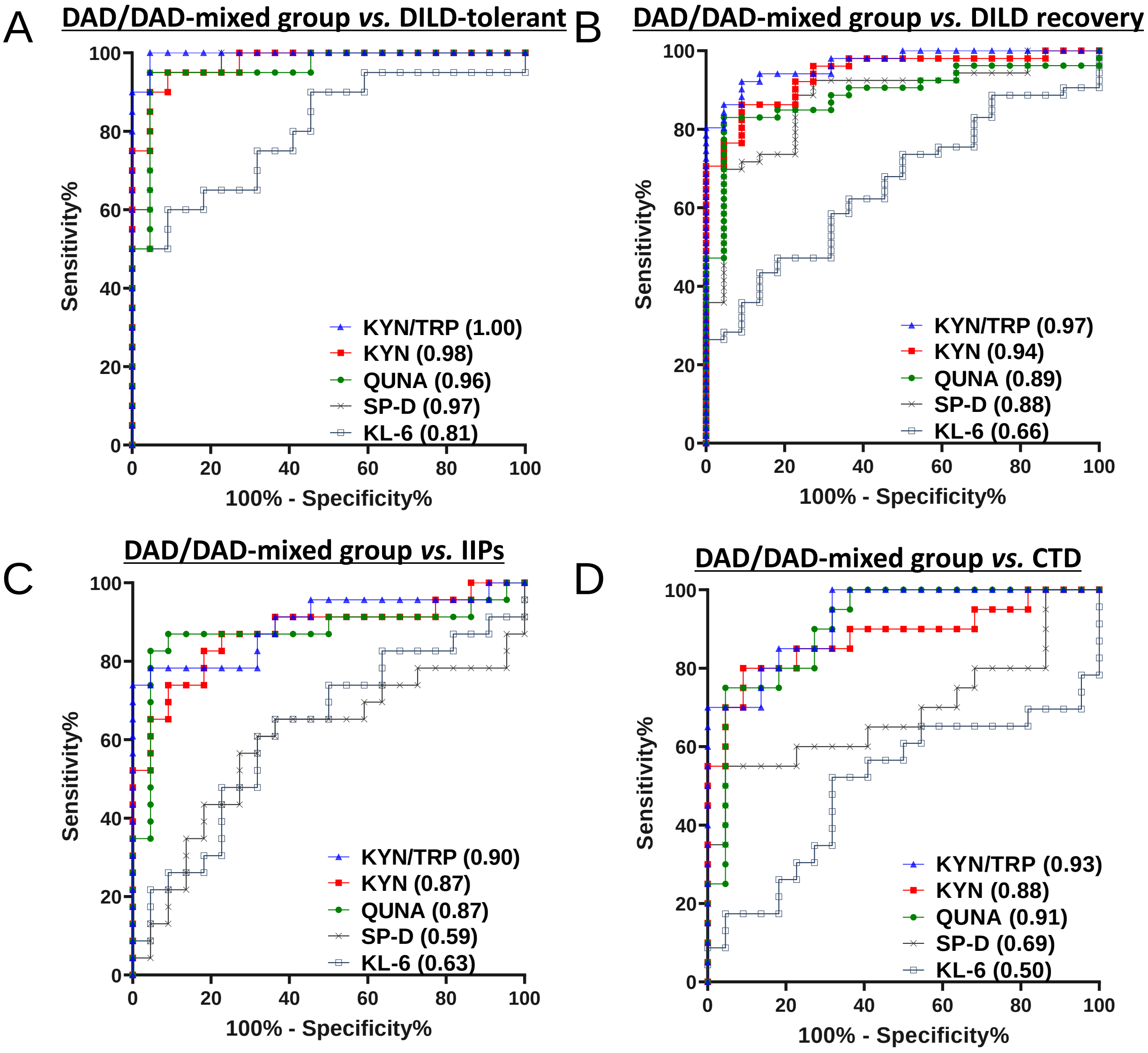

Figure S7. Diagnostic potentials of KYN, QUNA, KYN/TRP ratio, and conventional DILD biomarkers in DAD/DAD-mixed patients.

ROC curve analyses of serum levels of KYN and QUNA, KYN/TRP ratio, and levels of conventional ILD biomarkers (SP-D and KL-6) were performed between the groups using the quantitative data in the combined cohort. The ROC curves of DAD/DAD-mixed patients compared with DILD-tolerant **A**, DILD recovery **B**, IIPs **C**, or CTD **D** are shown. The values of AUROC are described in the parentheses of the labels for each tested biomarker. The AUROC values for other comparisons are summarized in Table 3.

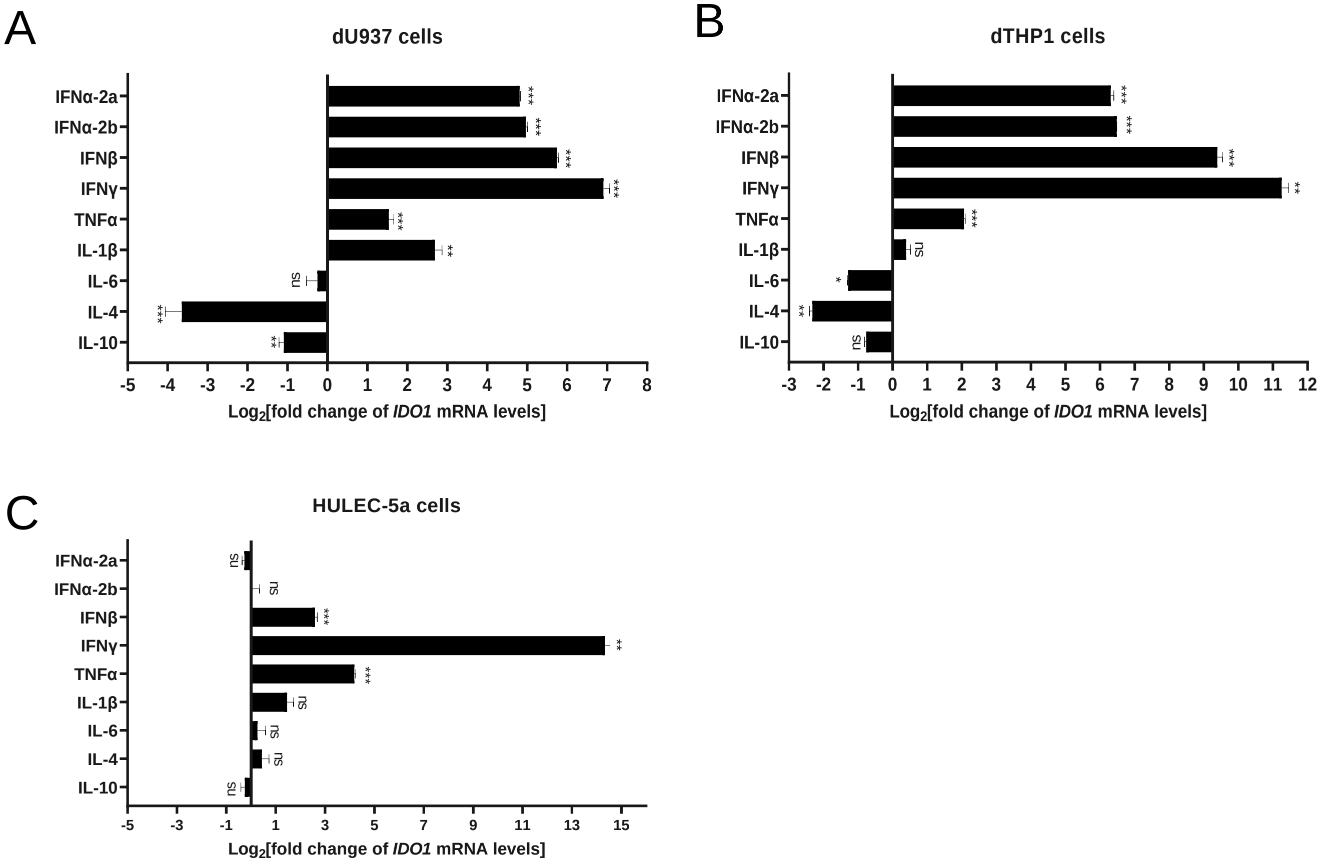

Figure S8. Effect of various inflammatory and anti-inflammatory stimuli on *IDO1* mRNA expression in differentiated macrophage cell lines and a lung endothelial cell line.

Fold changes of *IDO1* mRNA expression levels upon various inflammatory and anti-inflammatory stimuli (10 ng/mL for all cytokines) were examined in differentiated macrophage cell lines (**A** dTHP1 and **B** dU937) and lung ECs (**C** HULEC-5a). The error bar represents the mean ± standard deviation of three independent experiments. Statistical significance of *IDO1* mRNA levels between control cells and cytokine-treated cells was tested using Student's t-test with Bonferroni correction (ns, not significant; **, adjusted p-value < 0.01; ***, adjusted p-value < 0.001).

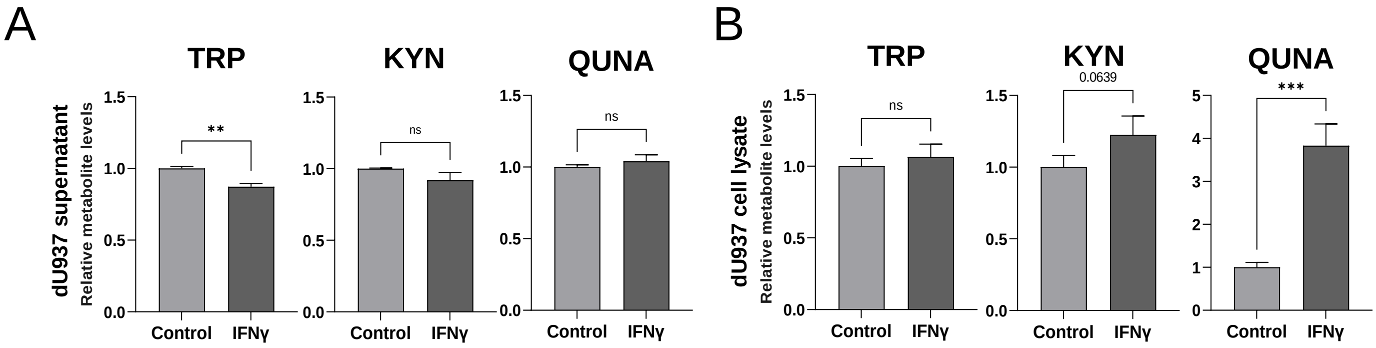

Figure S9. Induction of KYN pathway metabolites in cell lysates and supernatant of differentiated U937 cells treated with IFNγ.

Relative levels of TRP, KYN, and QUNA in supernatant **A** and whole cell lysate **B** of dU937 cells treated with 10% FBS-PBS (control) or IFNγ (10 ng/mL) for 24 h are shown. Each bar represents the mean ± standard deviation of three independent experiments. Statistical significance of mean values was tested using Student's t-test (ns, not significant; **, p-value < 0.01; ***, p-value < 0.001).

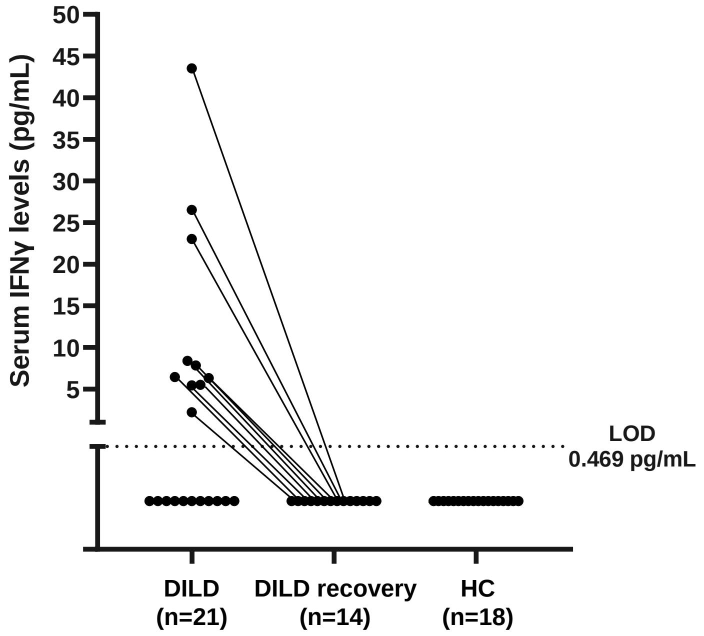

Figure S10. Serum IFNγ levels in DILD, DILD recovery, and HC.
Serum IFNγ levels in patients with DILD showing high serum KYN and QUNA levels (n=21), matched pair recovery samples (n=14), and HC (n=18) were measured using a commercially available sandwich ELISA kit. The matched-pair samples between the DILD and DILD recovery groups are indicated by solid lines. The limit of detection (LOD) was 0.469 pg/mL. It has been demonstrated that some patients with DILD showed detectable and elevated serum IFNγ levels, while their levels in all DILD recovery patients and healthy controls were below the detection limit.
