# Supplementary material for: Identification of kynurenine and quinolinic acid as promising serum biomarkers for drug-induced interstitial lung diseases
Source: Respir Res. 2024 Jan 14;25:31. doi: 10.1186/s12931-023-02653-6 (PMC10788992; doi:10.1186/s12931-023-02653-6)
Supplement: Supplementary file 4 — Additional file 4. Uncropped images of Western blotting analysis for IDO1 and GAPDH in dU937 and dTHP1 cells. [file 12931_2023_2653_MOESM4_ESM.pdf]

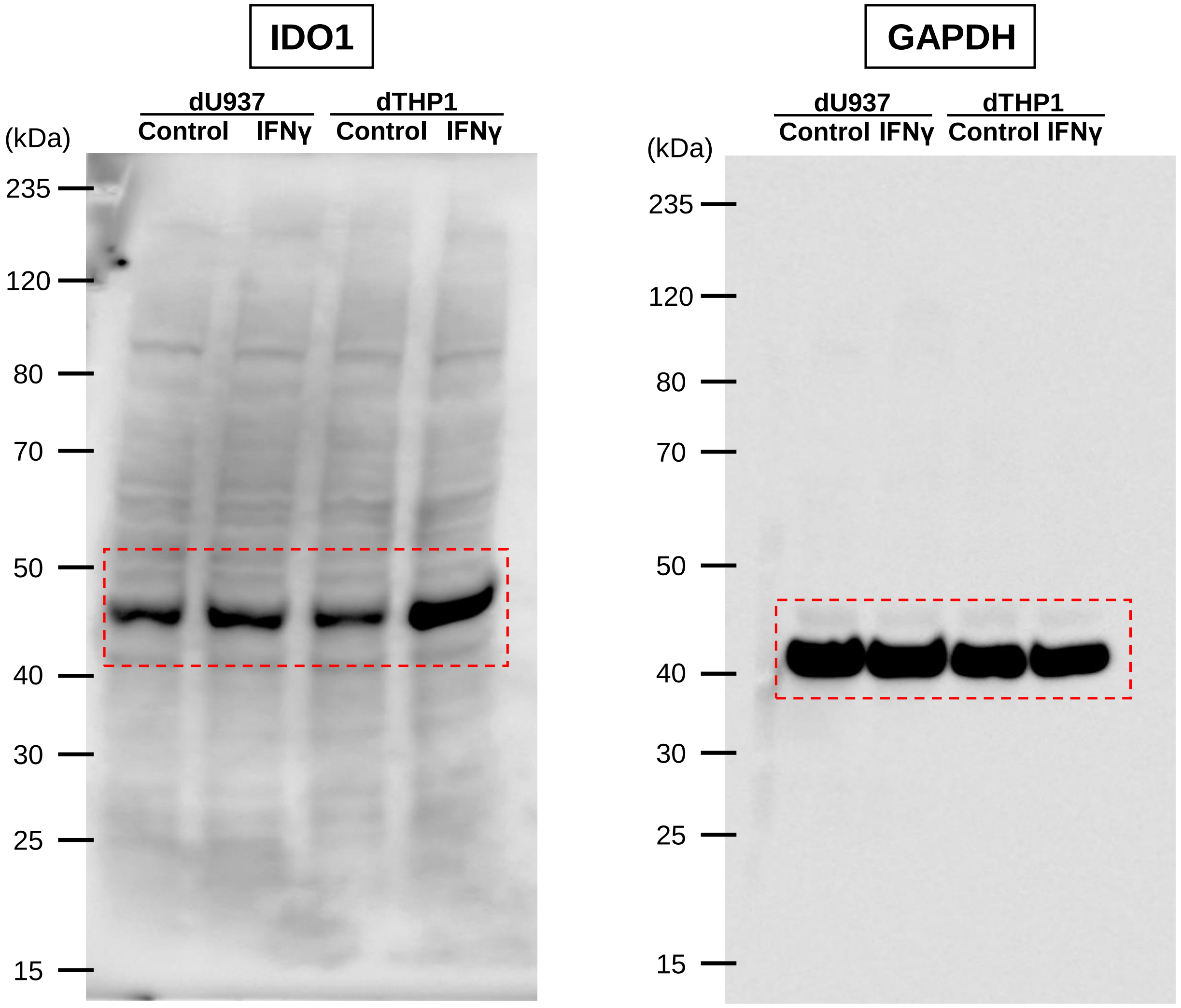

Uncropped images of Western blotting analysis for IDO1 and GAPDH in dU937 and dTHP1 cells. The red squares indicate the cropped images used in Figure 4E.
