# Supplementary material for: Identification of kynurenine and quinolinic acid as promising serum biomarkers for drug-induced interstitial lung diseases
Source: Respir Res. 2024 Jan 14;25:31. doi: 10.1186/s12931-023-02653-6 (PMC10788992; doi:10.1186/s12931-023-02653-6)
Supplement: Supplementary file 5 — Additional file 5. Uncropped images of Western blotting analysis for IDO1 and GAPDH in HULEC-5a cells. [file 12931_2023_2653_MOESM5_ESM.pdf]

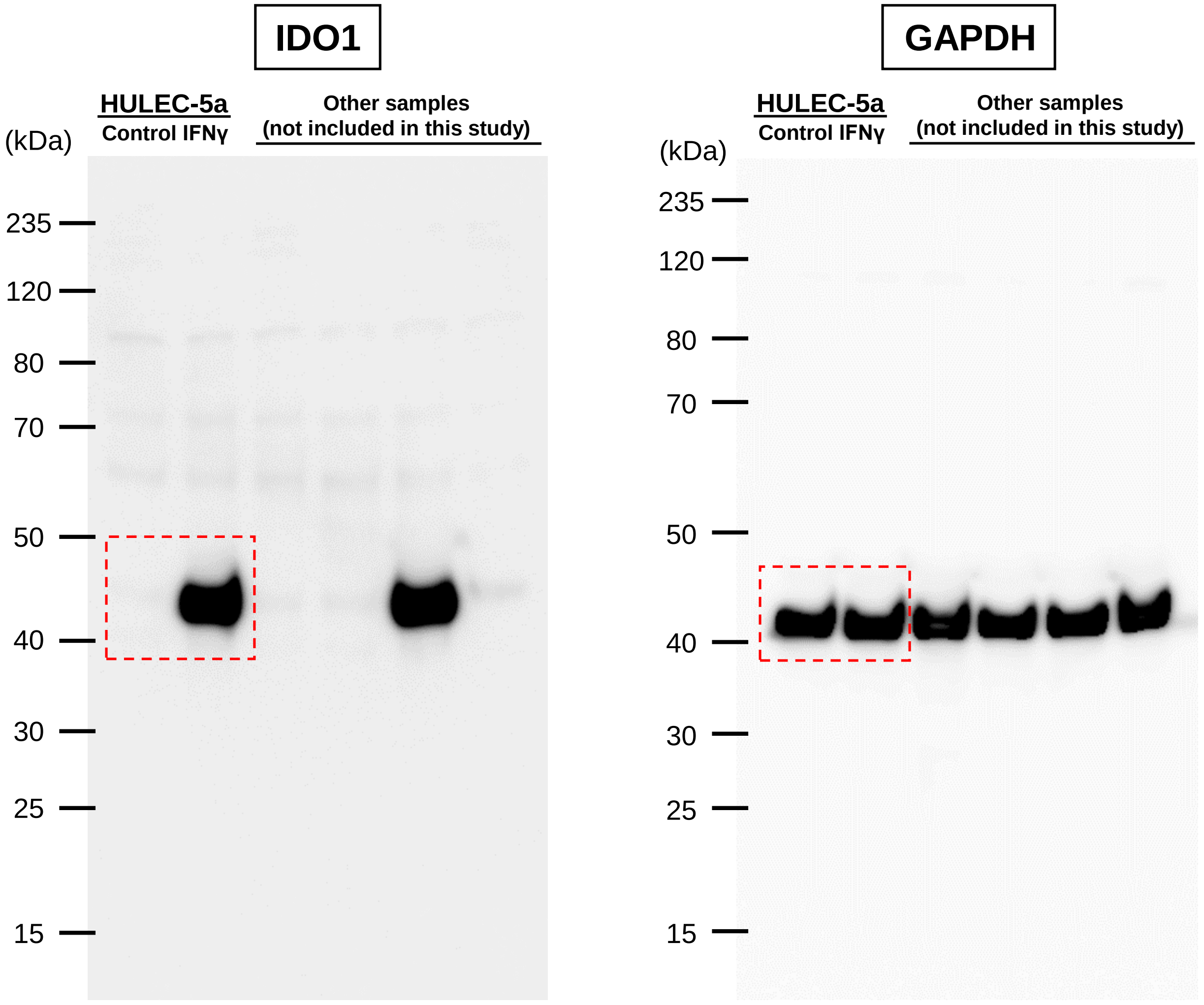

Uncropped images of Western blotting analysis for IDO1 and GAPDH in HULEC-5a cells.
The red squares indicate the cropped images used in Figure 5B.
